# Comparative genomic analysis of the arthropod muscle myosin heavy chain genes allows ancestral gene reconstruction and reveals a new type of 'partially' processed pseudogene

**DOI:** 10.1186/1471-2199-9-21

**Published:** 2008-02-06

**Authors:** Florian Odronitz, Martin Kollmar

**Affiliations:** 1 Abteilung NMR basierte Strukturbiologie, Max-Planck-Institut für Biophysikalische Chemie, Am Fassberg 11, D-37077 Göttingen, Germany

## Abstract

**Background:**

Alternative splicing of mutually exclusive exons is an important mechanism for increasing protein diversity in eukaryotes. The insect *Mhc *(myosin heavy chain) gene produces all different muscle myosins as a result of alternative splicing in contrast to most other organisms of the Metazoa lineage, that have a family of muscle genes with each gene coding for a protein specialized for a functional niche.

**Results:**

The muscle myosin heavy chain genes of 22 species of the Arthropoda ranging from the waterflea to wasp and *Drosophila *have been annotated. The analysis of the gene structures allowed the reconstruction of an ancient muscle myosin heavy chain gene and showed that during evolution of the arthropods introns have mainly been lost in these genes although intron gain might have happened in a few cases. Surprisingly, the genome of *Aedes aegypti *contains another and that of *Culex pipiens quinquefasciatus *two further muscle myosin heavy chain genes, called *Mhc3 *and *Mhc4*, that contain only one variant of the corresponding alternative exons of the *Mhc1 *gene. *Mhc3 *transcription in *Aedes aegypti *is documented by EST data. *Mhc3 *and *Mhc4 *inserted in the *Aedes *and *Culex *genomes either by gene duplication followed by the loss of all but one variant of the alternative exons, or by incorporation of a transcript of which all other variants have been spliced out retaining the exon-intron structure. The second and more likely possibility represents a new type of a 'partially' processed pseudogene.

**Conclusion:**

Based on the comparative genomic analysis of the alternatively spliced arthropod muscle myosin heavy chain genes we propose that the splicing process operates sequentially on the transcript. The process consists of the splicing of the mutually exclusive exons until one exon out of the cluster remains while retaining surrounding intronic sequence. In a second step splicing of introns takes place. A related mechanism could be responsible for the splicing of other genes containing mutually exclusive exons.

## Background

Alternative splicing is an important and widespread mechanism that is used by higher organisms to express molecularly distinct mRNAs in response to developmental and cellular contexts [1,2]. Mutually exclusive splicing, in which only one exon is chosen out of a cluster of alternative exons arranged in a tandem array, is a very frequent alternative splicing event on a genome-wide level [3,4]. Several mechanisms have been proposed that explain why only one of the two or more variants is included in the mature mRNA [5-7]. Mostly, Metazoa contain mutually exclusive exons only in pairs. Extreme cases for mutually exclusive splicing are the insects *Dscam *genes that have arrays of up to 52 variants as observed in the *Drosophila Dscam *gene [8]. A less dramatic example is the mutually exclusive spliced *Drosophila *muscle myosin heavy chain gene that can potentially produce 480 different mRNAs [9].

Myosins comprise a large superfamily of actin-based motors that fulfill a variety of cellular functions from cell division, cellular locomotion, and vesicle transport to muscle contraction [10,11]. 35 classes of myosins have been identified to date with each class being responsible for a different function [12-14]. The first myosin was identified in skeletal muscle tissue over hundred years ago (for a review about the history of muscle myosin see [15]) and, since different myosins turned up, it has been referred to as conventional myosin or class-II myosin. Class-II myosins comprise the largest and most extensively studied class not only because the muscle myosin genes and muscles have been in the focus of biophysical and biochemical studies for decades and because the metazoan species are the most studied organisms but also because this class contains the most isoforms per organism [12].

*Drosophila melanogaster *contains two class-II myosin genes, one encoding the muscle isoforms (*Mhc*) and one the nonmuscle isoform (*zipper*) [16]. The *Mhc *gene produces all different muscle myosins as a result of alternative RNA splicing [9]. This is in contrast to the organisms of most other taxa of the Metazoa lineage, that have a family of muscle myosin heavy chain genes with each gene coding for a protein specialized for a functional niche. For example, the nematode *Caenorhabditis elegans *expresses six muscle myosins [13], while the ascidian *Ciona intestinalis *genome contains five muscle myosin heavy chain genes [17] and vertebrate genomes encode up to 22 muscle myosin heavy chain isoforms [12].

The *Drosophila Mhc *gene consists of 30 exons including five clusters of alternatively spliced exons and one differentially included penultimate exon. Thus, 480 combinations of alternative exons are possible. The four clusters of alternative exons in the motor domain part of the gene code for 120 different variations of the motor domain. In contrast to the muscle myosins of the other metazoa species, changes modulating myosin function are thus limited to four regions in the head domain. These discrete regions of sequence variation have been shown to produce physiological differences among the various muscle types [18]. Although many variations are possible and all alternative exons get expressed at some point in *Drosophila's *life, only a limited number of combinations seem to be employed. For example, during *Drosophila *embryogenesis only seven *Mhc *transcripts have been found to be expressed [18].

The genome of *Drosophila melanogaster *was the third eukaryotic genome to be completely sequenced [19]. Since then, the number of sequenced organisms has increased rapidly. Of the phylum Arthropoda, the genomes of the mosquitos *Anopheles gambiae *[20] and *Aedes aegypti *[21] and the silkworm *Bombyx mori *[22] have been published, and 17 further insect genomes have been finished of which eleven belong to the *Drosophila *species group [23,24].

Originally, pseudogenes have been defined as DNA sequences that are derived from functional genes, but acquired such degenerative features as premature stop codons and frameshift mutations, which make them unable to produce functional proteins [25-27]. Non-processed pseudogenes are thought to result from tandem duplications of genes with subsequent accumulation of disabling mutations. Processed pseudogenes lack introns and their original upstream gene regulatory resions and presumably arise by retrotransposition of a mature messenger RNA (mRNA). While non-processed pseudogenes are commonly found near the functional original gene, processed pseudogenes are randomly inserted into the genome. Also, partially processed pseudogenes have been reported that sometimes contain the complete coding region [28,29]. Recent studies have shown, that pseudogenes are not just "Junk" DNA but often exhibit functional roles (for a review see [26]).

Here, we report the comparative genomic analysis of the muscle myosin heavy chain genes of all arthropod species that have completely been sequenced so far. On this basis we propose that the splicing process operates sequentially on the transcript involving the splicing of all unwanted alternative versions of an exon while retaining intronic sequence around the remaining variant.

## Results

### Identification and annotation of the muscle myosin heavy chains

**Figure 1:**
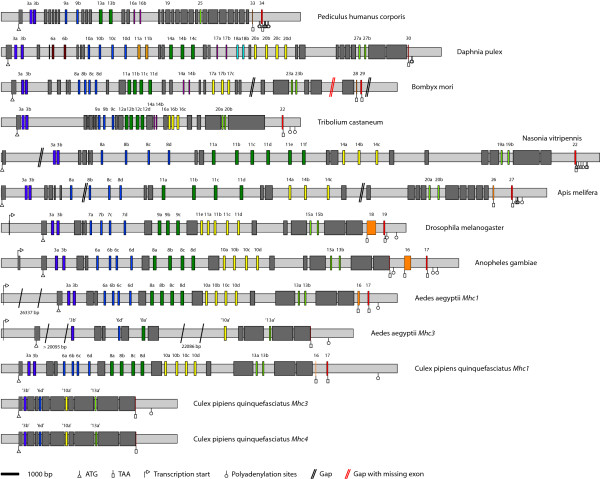
**Diagram of the arthropod *Mhc1 *genes with exon-intron structure**. The gene structures of the arthropod muscle myosins genes are shown using the following color code: light-gray: intron sequences; dark-gray: common exons; colored: alternatively spliced exons. The *Drosophila melanogaster Mhc1 *gene is shown as representative for all *Drosophila sp. Mhc1 *genes, because their gene structures only differ in the length of the introns. The transcriptional and translational start sites, the stop codons and polyadenylation sites are shown if they have been determined. Some genes are spread on several contigs. The corresponding gap positions are shown in black, if further exons are not expected, and in red, if exons are definitively missing. The genes are drawn to scale except for the *Aedes aegypti *genes where the extremely long introns have been shortened. Gaps have been filled with 100 bp although their exact length is unknown.

The arthropod muscle myosin heavy chain genes were identified by TBLASTN searches against the corresponding genome data of the different species using the *Drosophila melanogaster *protein as query (Figure 1, see Additional file 1). The species analysed were the mosquitos *Aedes aegypti, Culex pipiens quinquefasciatus *and *Anopheles gambiae*, the silkworm *Bombyx mori*, the honeybee *Apis mellifera*, the jewel wasp *Nasonia vitripennis*, the waterflea *Daphnia pulex*, the rust-red flour beetle *Tribolium castaneum*, the body louse *Pediculus humanus corporis*, and thirteen *Drosophila *species (Table 1). According to the general nomenclature for myosin sequences [12] the alternatively spliced muscle myosin heavy chain genes are named *Mhc1*, and the non-muscle myosin heavy chain genes are denoted *Mhc2*. The sequences were assigned by manual inspection of the genomic DNA sequences. Exons have been confirmed by the identification of flanking consensus intron-exon splice junction donor and acceptor sequences (Figure 1) [30]. Because of the five to nine clusters of mutually exclusive exons and the included or excluded penultimate exon, automatic identification of all exons failed. The genomic sequences of *Apis mellifera *and *Bombyx mori *contain several gaps that at least in one case must have contained missing exons. The expression of the myosin genes including the transcription of some of the mutually exclusive exons has been confirmed by analysis of corresponding EST data.

**Table 1:** Nucleotide ID's and number of combinations of alternative exons for the motor domains and the full-length proteins.

Species	Species Abbr.	Nucleotide ID's GenBank:	Motor domain	Full-length protein
*Daphnia pulex*	*Dap*		1536	> 3072
*Bombyx mori str. Dazao*	*Bm*	AADK01001734, BAAB01137479 BAAB01017092, AV404226 AADK01040535, AADK01049792	192	768
*Tribolium castaneum str. Georgia GA2*	*Tic*	AAJJ01000118	192	> 384
*Nasonia vitripennis str. SymAX*	*Nav*	AAZX01008059, AAZX01007288	144	> 288
*Apis mellifera str. DH4*	*Am*	AADG05005753, AADG05005754 AADG05005757	96	384
*Drosophila ananassae TSC#14024-0371.13*	*Da*	AAPP01015693	120	480
*Drosophila erecta TSC#14021-0224.01*	*Der*	AAPQ01007075	120	480
*Drosophila grimshawi TSC#15287-2541.00*	*Dg*	AAPT01021775	120	480
*Drosophila hydei*	*Dh*	X77570	120	480
*Drosophila melanogaster*	*Dm*	NM_165190	120	480
*Drosophila mojavensis TSC#15081-1352.22*	*Dmo*	AAPU01010481	120	480
*Drosophila persimilis MSH-3*	*Drp*	AAIZ01000908, AAIZ01000907 AAIZ01000906, AAIZ01000905 AAIZ01000904, AAIZ01024863 AAIZ01000903	120	480
*Drosophila pseudoobscura MV2-25*	*Dp*	AAFS01000199	120	480
*Drosophila sechellia Rob3c*	*Dse*	AAKO01001629	120	480
*Drosophila simulans str. white501*	*Dss*		120	480
*Drosophila virilis TSC#15010-1051.87*	*Dv*	AANI01016210, AANI01016211	120	480
*Drosophila yakuba Tai18E2*	*Dy*	AAEU01002444, AAEU01002445 AAEU01002446	120	480
*Drosophila willistoni TSC#14030-0811.24*	*Dw*	AAQB01006734	120	480
*Anopheles gambiae str. PEST*	*Ang*	AAAB01008980	128	768
*Aedes aegypti str. Liverpool Mhc1*	*Aea*	AAGE02009209	128	512
*Aedes aegypti str. Liverpool Mhc3*	*Aea*	AAGE02009019, AAGE02009018	1	1
*Pediculus humanus corporis str. USDA*	*Pdc*	AAZO01001178	16	32
*Culex pipiens quinquefasciatus JHB Mhc1*	*Cpq*	AAWU01000999	128	512
*Culex pipiens quinquefasciatus JHB Mhc3*	*Cpq*	AAWU01000999	1	1
*Culex pipiens quinquefasciatus JHB Mhc4*	*Cpq*	AAWU01000999	1	1

The untranslated first exons of the genes have been assigned by analysing EST data, if possible. Because untranslated 5' exons were found for all those species for which EST data covering the amino-termini of the genes is available, it is expected that the other arthropod myosin genes also contain untranslated first exons. Accordingly, the unambiguously identified exons have been numbered starting with exon two. Duplicated exons were named in alphabetical order according to the direction of transcription, the exception being the alternatively spliced exon 11 of the *Drosophila *Mhc1 of which the first of the mutually exclusive exons was named 11e for historical reasons [9]. The differentially included penultimate exons of the *Drosophila *species have been predicted based on their similarity at the DNA level. Although this exon mainly consists of untranslated bases and its identity between the *Drosophila *species is almost as low as that found in intron regions, the exon borders are conserved enough to be recognised. The carboxy-terminal exons of the other arthropod *Mhc1 *genes have been confirmed by analysing EST data, if possible. For *TicMhc1 *and *DapMhc1 *only one carboxy-terminal exon could be confirmed by EST data. However, given the exon conservation between all arthropod *Mhc1 *genes it is expected that both genes contain another carboxy-terminal exon. For *Nasonia*, EST data is not available. The carboxy-terminal exon of the *NavMhc1 *gene was identified based on its homology to the other *Mhc1 *exons. An exon corresponding to the penultimate exon of the other genes could not be identified.

The *Drosophila sp. Mhc1 *genes, the *AeaMhc1 *and the *CpqMhc1 *gene contain consensus polyadenylation signals AATAAA, while the *Mhc1 *genes of *Ang*, *Am*, *Dap*, *Nav*, *Pdc*, and *Tic *contain polyadenylation signals of type AAAAAA. For the *DmMhc1 *gene it has been shown that the use of either polyadenylation site is not regulated [31,32] and the same might be true for the two or multiple polyadenylation sites of the other arthropod genes.

### Identification of further muscle myosin heavy chain genes in *Aedes aegypti *and *Culex pipiens quinquefasciatus*

Surprisingly, a second muscle myosin heavy chain gene has been identified in *Aedes aegypti *(Figure 1) and named *Mhc3*. The *Mhc3 *gene contains the same exon organisation as *Mhc1 *except that it does not have any cluster of alternatively spliced exons and misses the two carboxy-terminal exons (Figure 1). Many EST clones provide supporting evidence for the deduced carboxy-terminus, the amino-terminal untranslated exon1, and other parts of the gene. The exons related to the alternatively spliced exons of *Mhc1 *are either identical ("exon3b") or very similar to one of the *Mhc1 *exons. The protein sequence of Mhc3 has an overall sequence identity of 91.4% to Mhc1. Besides the different carboxy-termini, the largest differences are in loop-1, which is three residues shorter in Mhc3, and in loop-2, which has only six instead of ten glycines and might therefore be structurally more restricted. The *Culex pipiens quinquefasciatus *genome encodes another two muscle myosin heavy chain genes that are very similar to each other and have been named *Mhc3 *and *Mhc4 *(Figure 1). Both have the same exon organisation as the *CpqMhc1 *gene except that they do not have any cluster of alternatively spliced exons and miss the two carboxy-terminal exons. Another difference is that alternative exons 8 are fused to the following constitutive exons in the *Mhc3 *and *Mhc4 *genes. The protein sequence identity between *CpqMhc3 *and *CpqMhc4 *is 92.0%, the identity to *CpqMhc1 *is 84.4% and 90.4%, respectively. Surprisingly, *AeaMhc3*, *CpqMhc3 *and *CpqMhc4 *retained identical variants of the alternatively spliced exons of the corresponding *Mhc1 *genes.

### The *BmMhc1*, *TicMhc1*, *PdcMhc1 *and *DapMhc1 *genes contain further clusters of alternatively spliced exons

The analysis of the *BmMhc1*, *TicMhc1*, *PdcMhc1*, and *DapMhc1 *genes revealed further clusters of alternatively spliced exons compared to the *DmMhc1 *gene. All further sets of alternative exons encode for sequence that is part of the motor domain. The additional alternative exon of *Bm*, *Pdc *and *Tic *is conserved between these three organisms, and is also encoded within the *Dap Mhc1 *gene. It is located between the alternatively spliced exons 11 and 17 (*Bm*), alternative exon 13 and constitutive exon 19 (*Pdc*), and alternative exons 12 and 16 (*Tic*), respectively, and separated from the neighbouring alternatively spliced exons by constitutively expressed exons (Figure 1). In contrast to the other alternatively spliced exons, these alternatively spliced exons are different in length and amino acid conservation (see Additional file 2, figure S6A). The first part of the exon encodes part of loop-2 (see below), that is a very flexible loop involved in actin-binding. In the arthropod genes it mainly consists of glycines, arginines, and lysines. Thus, the alternatively spliced exons of *Bm*, *Tic*, *Pdc*, and *Dap *encode different numbers and compositions of these residues. The second part of the alternatively spliced exon is part of the following alpha-helix and hence completely conserved in length and strongly conserved in composition. In addition to this cluster of alternatively spliced exons, the *DapMhc1 *gene contains three further sets of alternatively spliced exons extending its number of clusters of alternatively spliced exons to nine (compared to five in *Drosophila*). Alternative exon 6 encodes an alternative P-loop to loop-1 sequence, alternative exon 11 directly follows the alternative exon encoding a structural part near the ATP-binding site, and alternative exon 18 encodes an alternative version of the sequence after loop-2 (Figure 1).

### The *PdcMhc1 *gene encodes a strongly reduced set of possible transcripts

The *Pediculus humanus corporis Mhc1 *gene contains the most reduced set of alternative exons (Figure 1). It has four sets of alternative exons each comprising two variants. However, the sequence encoding part of the converter domain, which is encoded by sets of three to five alternative exons in the other arthropod genes, has been fused to the following exon forming one constitutive exon in the *PdcMhc1 *gene (exon 19, Figure 1). Also, the part in the tail domain encoded by a set of two alternative exons in all other arthropod genes is represented by only one exon in the *PdcMhc1 *gene (exon 25). Altogether, the alternative exons encode for 16 different versions of the motor domain and 32 different mRNAs of the *PdcMhc1 *gene, compared to potentially 120 different combinations of alternative exons for only the motor domain of the *Drosophila Mhc1 *gene.

### Conservation of alternatively spliced exons

**Figure 2:**
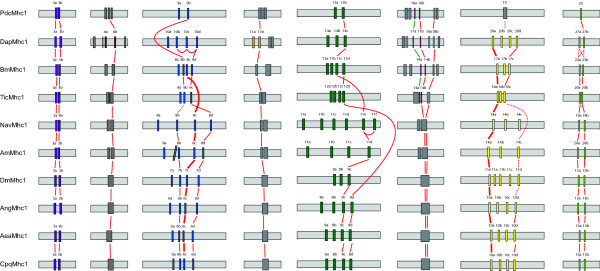
**Relationships between alternatively spliced exon**. Sections of the *Mhc1 *genes of Figure 1 have been aligned showing the relationship between the exon-intron structures of the regions containing alternatively spliced exons. Continuous lines connect variants that are almost identical and thus expected to be derived from a common ancestor. Bold lines connecting alternative exons in regions containing multiple variants per *Mhc1 *gene highlight particularly conserved exons in these sets. Dotted lines represent putative connections between certain variants although their identity is not very strong on the protein level.

**Figure 3:**
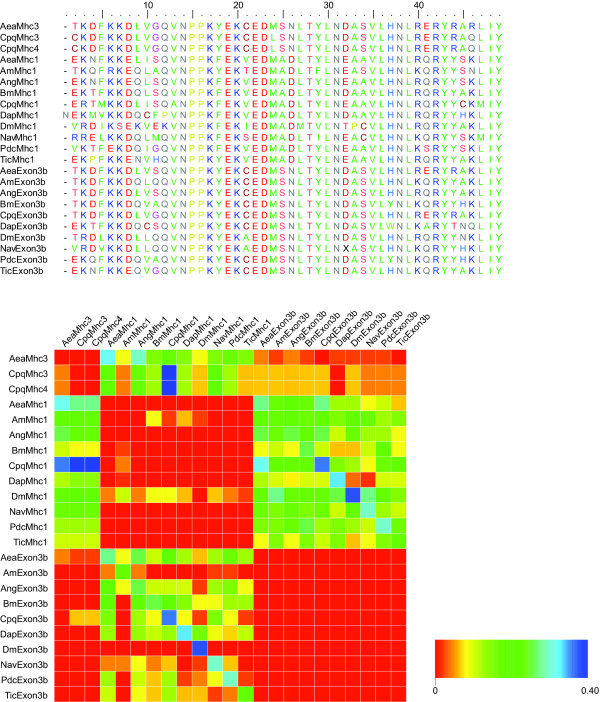
**Sequence conservation in the first set of the alternatively spliced exons**. On top, the protein sequence alignment of the alternative exons is shown. The upper sequences, termed Mhc1, Mhc3, and Mhc4, respectively, represent the variant "a" exons. Below, the comparison of the sequence identity between each exon and variant "a" and "b" of every other Mhc1 protein is shown. The graphic has to be read in columns. The higher identity between an exon listed on top and variant "a" or "b" of a certain Mhc1 protein listed on the left side has been set to 1 (red color) while the difference of the lower identity to the value of the higher identity is plotted for the other combination of exons. Thus, in every column the higher identity of the named exon to one of the variants of the other Mhc1 proteins is visualized.

The number of variants differs between the arthropod species for many of the alternatively spliced exons (Figures 1 and 2). For the first set of alternatively spliced exons two variants have been found in all *Mhc1 *genes. Both differ by two absolutely conserved residues, namely the amino acids alanine and aspartate at positions 25 and 26 in the "a" variants of the exon that are substituted by serine and asparagine in the "b" variants (Figure 3). A slightly less conserved marker for the "b" variants is a cysteine at position 21. Variant 3a of the *DapMhc1 *is an exception as it has an additional residue at the N-terminus compared to the other *Mhc1 *variant "a" exons. The *DapMhc1 *gene encodes three clusters of alternatively spliced exons not found in the other arthropod *Mhc1 *genes. For all three clusters exons variant "b" is more homologous to the corresponding amino acid sequences of the other Mhc1 proteins than variant "a" (see Additional file 2, figures S2, S4, and S6B). The alternatively spliced exons of *BmMhc1*, *DapMhc1*, *PdcMhc1 *and *TicMhc1 *covering loop-2 are different in length and starting position (see Additional file 2, figure S6A). However, the "a" variants are more similar to each other than to the "b" variants and the corresponding amino acid sequences of the other Mhc1 proteins. Thus, the common ancestor of *Bm*, *Dap*, and *Tic *had in all probability already contained an "a" and a "b" variant. Completely conserved residues characterizing the "a" variant are a serine at the end of loop-2, a glutamate at position 3, and a leucine at position 8 of the following helix ([G/K/R 8-9]**S **[G/A]F [**Q**/M]TVS [S/A]**L**YR). Except for *PdcMhc1*, all arthropod *Mhc1 *genes have two variants of the mutually exclusively spliced exon in the tail (Figure 2; see also Additional file 2, figure S8). The most conserved differences between the two variants are an aspartate at position 14 in variant "b" (either an asparagine or a glutamine in variant "a") and an asparagine at position 24 (an arginine in variant "a"). In addition, at position 15 the "b" variants have a large hydrophobic residue (leucine, methionine, or phenylalanine) while the "a" variants have a small polar residue (serine or threonine). In contrast to the other *Mhc1 *genes, the "a" variant of *DapMhc1 *is closer related to the "b" variants than to the other "a" variants.

The situation is more complex for the remaining clusters of mutually exclusive exons that contain three to six variants. The exon encoding a loop-helix motif adjacent to the ATP-binding site (blue color in Figure 1) is not as conserved as the other alternatively spliced exons (Figure 2; see also Additional file 2, figure S3). Therefore, it is difficult to identify characteristic residues/motifs for the respective variants. Except for the *PdcMhc1 *and *TicMhc1 *genes all genes contain four variants. The variant with the most characteristic residues is variant "c". It is characterized by a positively charged residue at position 8 (arginine or histidine), a conserved arginine at position 21, and a conserved asparagine at position 26. None of these residues appear in any of the other variants at the respective positions. The *TicMhc1*, *PdcMhc1*, and *DapMhc1 *genes have lost this variant. The only strong characteristic of variant "d" is a conserved isoleucine or valine at position 20 that is found in all *Mhc1 *genes. Variants "a" and "b" do not contain any distinguishing residues.

The alternatively spliced exons spanning the relay helix and the relay loop are the longest and most conserved of the mutually exclusive exons (see Additional file 2, figure S5). The variability ranges from two variants in the *Pediculus Mhc1 *gene to six variants in the *Nasonia *gene (Figures 1 and 2). The least conserved part of the exon is the relay loop that is not embedded in the motor domain. In this region, characteristic residues for certain variants are found. Variant "c" is characterized by a conserved glutamine at position 49 and either a glutamine or an asparagine at position 50. A copy of this variant is present in all *Mhc1 *genes except that of *Tic*. Another conserved variant is variant "d" characterized by a glutamine at position 49 followed by a proline at position 50. This variant appears in the *Mhc1 *genes of *Aea*, *Ang*, *Cpq*, *Tic*, and *Bm*. Similar to the situation for the alternatively spliced exon at the ATP-binding site, the other variants are not conserved enough to define characteristic residues. It is thus not clear which were present in the ancient arthropod gene and which arose through exon duplication in the individual genes. Again, the *DapMhc1 *is the exception because its first two variants, characterized by two conserved methionines at positions 42 and 55, differ from all other variants.

The variants of the cluster of alternative exons encoding part of the converter domain also show a high degree of variability (Figure 2; see also Additional file 2, figure S7). Two of the variants have characteristic features. Variant "a" is the most conserved of the variants at the protein level having a conserved methionine at position 9 and a conserved cysteine at position 26. These residues do not appear in any of the other variants of this cluster. Variant "a" of this cluster is conserved in the *Mhc1 *genes of all species and therefore must have been present in their common ancestor. The last of the variants has a characteristic feature at the DNA level. The intron following the last variant always has a GT 5' splice site. This is in contrast to all other variants of this exon whose following introns have a GC 5' splice site. At the amino acid level this variant is characterized by a lysine at position 2, a cysteine at position 5 and a glutamate at position 20.

Wherever EST and/or cDNA data was available a differentially excluded penultimate exon could be identified. These exons are very short (one to thirteen residues) and not conserved (see Additional file 2, figure S9), and therefore similar exons have not been predicted for the species for which EST data is not available. For *Ang *three carboxy-termini have been identified. Based on EST data the *AngMhc1 *transcript may also end with a short extension to the antepenultimate exon. This C-terminus is similar to that found for *AeaMhc3 *and *CpqMhc4 *and might be used in a similar combination of the other alternatively spliced exons.

### Phylogenetic analysis of the arthropod muscle myosin heavy chain genes

**Figure 4:**
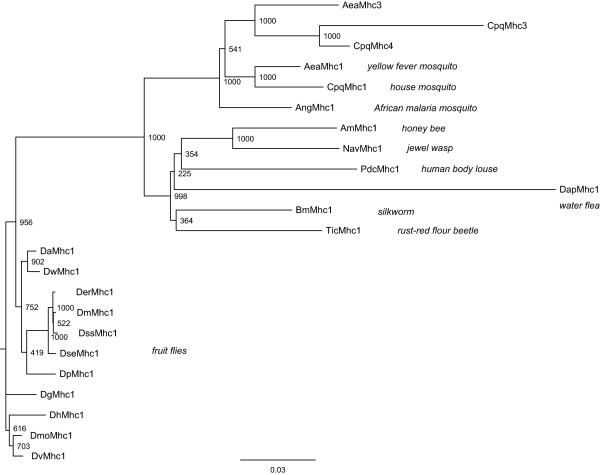
**Phylogenetic tree of the arthropod muscle myosin heavy chain proteins**. The amino acid sequences of the full-length proteins were aligned manually. Because of their incompleteness the sequences of *Drosophila persimilis *and *Drosophila yakuba *have been omitted from the tree calculation. Support values for each internal branch were obtained by 1,000 bootstrap steps. The scale bar corresponds to 0.1 estimated amino acid substitutions per site.

A phylogenetic tree of all arthropod Mhc1 protein sequences, always incorporating the first variant of the clusters of alternatively spliced exons and excluding the differentially included penultimate exon, has been generated (Figure 4). In general, the tree reflects the phylogenetic relationship between the species. The *Aea*Mhc3 sequence is most closely related to the *Cpq*Mhc3 and the *Cpq*Mhc4 sequence implicating that the last common ancestor of *Aedes *and *Culex *already had one of these genes. The phylogeny of the *Drosophila *species slightly differs compared to other analyses [23]. Thus, the *Da*Mhc1 sequence would have been expected to separate after the divergence of the *Dp*Mhc1 sequence. Similarly, the *Dse*Mhc1 gene would have been expected to be the closest relative of the *Dss*Mhc1 sequence. Overall, the sequence identity is very high. Between *Dap*Mhc1 and the other sequences the identity is 70.6 – 77.9%, while it is between 77.0% and 99.7% between the other species.

### Predicting the gene structure of an ancient *Mhc1 *gene

**Figure 5:**
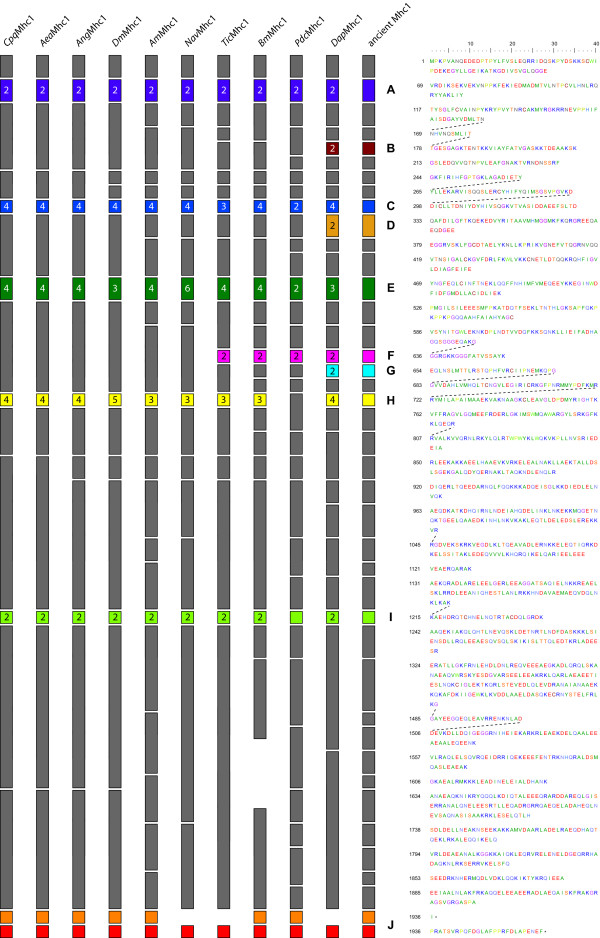
**Diagram of the arthropod Mhc1 proteins**. The exon-intron structure of the *Mhc1 *genes is shown based on the protein sequence. Exons are shown as boxes while introns are represented by spaces. The same colour scheme has been used as in Figure 1. Numbers on alternative exons denote the number of variants. The exons are drawn that the intron positions align between the different *Mhc1 *genes. Thus, the exon lengths are not drawn to scale (e.g. the exons encoding the variable loop-2 are different in lengths). On the right side, the protein sequence of *Drosophila melanogaster *Mhc1 is shown as reference. Dotted lines connect amino acids that are derived from split codons.

Whenever intron positions are shared between the genes, the corresponding type of splice site is conserved, with the exception of the shared exon 9 (*AmMhc1*), exon 10 (*TicMhc1*), exon 9 (*BmMhc1*), and the alternatively spliced exon 11 of *DapMhc1 *(Figure 5). All introns have consensus dinucleotide borders except those downstream of the last variant of the cluster of alternative exons encoding part of the motor domain (homologs of exon 11 in *DmMhc1*), which have a GC dinucleotide at the 5' donor site instead of the consensus GT. The 3' exons of these alternatively spliced exons again have a consensus GT site. Exon '10a' of *AeaMhc3 *is almost identical to exon 10a of *AeaMhc1 *and the following intron also has a GC dinucleotide at the 5' donor site. In contrast to the introns following the exons 9 of *AmMhc1*, *NavMhc1*, and *BmMhc1*, and the intron following exon 10 of *PdcMhc1 *that have a consensus GT site, exon 10 of *TicMhc1 *has a GC 5' donor site. The intron following exon 11a of *DapMhc1 *starts with a consensus GT site, while the intron following exon 11b starts with the absolutely rare GA dinucleotide. Also, all split codons are shared between the genes.

In the part encoding the motor and the neck domain, all intron positions are shared by at least two genes (Figure 5). In the coiled-coil tail domain, all genes have lost several introns so that the exons are considerably longer and the intron positions in many cases are not identical. Assuming, that introns have in most cases been lost and were not gained during evolution [33], an ancient arthropod *Mhc1 *gene can be reconstructed (Figure 5). The ancient *Mhc1 *gene is expected to contain all intron positions that appear in at least one of the analysed *Mhc1 *genes. In the motor domain, the proposed ancient *Mhc1 *gene structure completely resembles the *DapMhc1 *gene. The exon lengths are between 30 and 210 bp. The exons in the tail domain are considerably longer (up to 480 bp).

### Structural implications of the alternatively spliced exons

**Figure 6:**
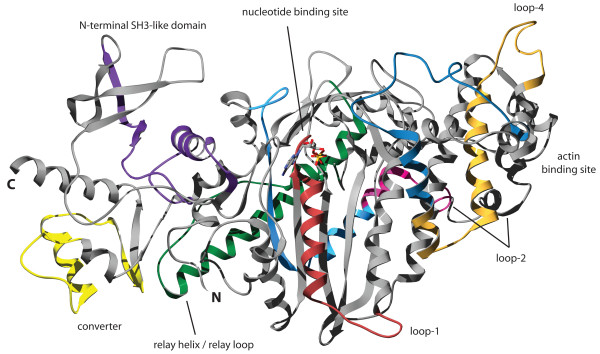
**Structure of the myosin motor domain**. The structure of the motor domain of the class-II myosin of *Dictyostelium discoideum *has been used to highlight the regions encoded by alternatively spliced exons in arthropod *Mhc1 *genes. The color-coding is the same as in Figure 1 allowing the identification of corresponding regions.

The locations of the alternatively spliced exons of *DmMhc1 *in the motor domain have been discussed in detail elsewhere [34]. The position of the additional alternatively spliced exons of the *BmMhc1*, *TicMhc1*, *PdcMhc1*, and *DapMhc1 *genes in the structure of the motor domain are shown in Figure 6. The alternative exons of *DapMhc1 *encoding the structural part from the P-loop to loop-1 have identical P-loop sequences. The loop-1 sequences are identical in length but differ significantly in composition. Studies have shown that the flexibility of this loop affects the rate of ADP and phosphate release, with greater flexibility leading to an enhancement in the rate of product release [35]. Although the amino acid composition is different between the alternative variants, both contain two glycines and a similar overall charge. The alternative exons of *DapMhc1 *including loop-4 are similar in length and composition. This region of the motor domain has not been investigated so far and therefore functional consequences of differences in the two variants cannot be drawn. Loop-4 has been postulated to be important for the proper localization of class-I myosins that contain elongated loops that sterically interact with actin-binding proteins [36] but the loop-4 sequences are almost identical between the two *DapMhc1 *variants and the two variants must therefore modulate a different property of the motor domain. The loop-2 sequence is modulated by alternative exons in the *BmMhc1*, *DapMhc1*, *PdcMhc1*, and *TicMhc1 *genes. By studies of the *Dictyostelium *class-2 myosin with its loop-2 replaced with the analogous loop from four other myosins with different enzymatic activities, loop-2 was shown to be involved in the weak and the strong binding interactions with actin [37]. It also plays an important role in the rate-limiting step of P_i _release [38,39]. The exon variants of the *BmMhc1*, *DapMhc1*, *PdcMhc1*, and *TicMhc1 *genes encoding the loop-2 sequence have identical numbers of lysine and arginine residues. The "a" variants are always one residue shorter and have only four instead of five glycines. These differences are, however, very subtle and their influence on actin binding is expected to be very small. The variants of the alternative exon in *DapMhc1 *following loop-2 are very similar. This part of the motor domain has also not been investigated so far.

## Discussion

25 muscle myosin heavy chain genes have been identified in 22 species of the Arthropoda. All sequences share strong homology to the alternatively spliced *Mhc1 *gene that was first described in *Drosophila melanogaster *[9]. The genes contain five to nine clusters of mutually exclusive exons and an penultimate exon that might either be included or excluded in the mRNA, and were assigned by manual inspection of the genomic DNA sequences (Figure 1). Because of the many clusters of alternatively spliced exons automatic identification of all exons failed. This is probably also the main reason for the wrong prediction of the exon organisation of the Anopheles *Mhc1 *gene (supplementary material of [20]).

Altogether, alternative splicing of *Mhc1 *transcripts could result in several hundred differently spliced mRNAs (Table 1). The *Pediculus Mhc1 *gene has the least alternatives for its alternatively spliced exons resulting in a theoretical maximum of 32 different mRNAs, while the water flea gene could result in at least 3072 different mRNAs. Thus, except for *Pediculus*, *Nasonia*, and *Apis mellifera *all arthropod *Mhc1 *genes, for which all exons could be identified, outscore the 480 mRNA possibilities of *Drosophila melanogaster*. Although the number of possible transcripts seems vast compared to the number of different muscle myosin heavy chain genes in other metazoa species, the regions to modulate the function of the protein are limited to five to nine. In *Drosophila melanogaster*, all alternative exons are expressed depending on the developmental stage, but only a limited number of combinations seem to be employed [18]. Whether all alternative exons are expressed in the other arthropod species and which combinations are used has yet to be determined.

The phylogenetic analysis of the Mhc1 protein sequences agrees with the expected phylogenetic relationship between the species. There are two notable exceptions in the *Drosophila *species section of the tree. The *Dse*Mhc1 sequence would have been expected to be the closest relative of the *Dss*Mhc1 sequence, and the *Da*Mhc1 sequence would have been expected to separate after the split of the *Dp*Mhc1 and *Drp*Mhc1 sequences. There are two possible ways to explain this observation. Either, the *Mhc1 *genes have evolved asynchronously as has been found for many yeast genes [40] or the genes might have incorporated back-mutations. The sequence identities of 96.1 to 99.7% are very high, and thus only a few mutations would lead to a different phylogenetic classification.

The *Tribolium castaneum*, *Pediculus humanus corporis*, and *Bombyx mori Mhc1 *genes contain one additional and the *Daphnia pulex Mhc1 *gene contains four additional clusters of alternatively spliced exons compared to the *Drosophila melanogaster *gene (Figure 1, Figure 2). All additional alternatively spliced exons are mutually exclusive and encode parts of the motor domain. The additional exons of the *Tic*, *Pdc*, and *Bm Mhc1 *genes encode alternative versions of the loop-2 sequence while the additional exons of the *Dap Mhc1 *gene are spread over the entire motor domain. In each case, the 3' variant is more homologous to the corresponding sequences in the other *Mhc1 *genes than the 5' variant (Figure 2).

A similar conservation is found for alternative exons with multiple variants (Figure 2). In almost all cases, the most 3' variant is the most conserved one. Of the cluster of alternative exons encoding part of the motor domain near the ATP-binding site (exon 7 in *DmMhc1*), the last of the variants is the only variant that is conserved in all species. The other variants are either missing in certain species, or are very similar to each other as well as to those of other species, so that it is not clear whether they have been derived from independent variant duplications or whether they were present in a common ancestor. Thus, all variants except for the most 3' variant evolved after the separation of *Daphnia *from the other species. The variants encoding the relay-helix and the relay-loop are highly conserved. Conserved differences confine to only one or two residues. The penultimate of the variants seems to be the most conserved, although mutation of one residue might change this. The exon encoding part of the converter domain has two highly conserved variants, the most 5' and the most 3' variants. The most 3' variant is distinguished from all other variants of this set of alternative exons at the DNA level because the following intron starts with a GT donor site. The most 5' exon is the most important, though not the only, determinant for flight capabilities [41,42].

Based on the exon-intron patterns of the 21 *Mhc1 *genes the gene structure of the ancient arthropod *Mhc1 *gene can be predicted. The prediction is based on the assumption that it is very unlikely that the different species, distributed over a broad taxonomic range, invented introns at the same positions independently from each other. In the first half of the genes encoding the motor and the neck domain, all intron positions are shared by at least two genes (Figure 5). The exons encoding the coiled-coil tail domain starting at amino acid 850 are considerably longer and the intron positions in almost all genes are not identical. It is highly probable that further sequencing of arthropod *Mhc1 *genes will reveal different exon-intron patterns in the tail region while intron positions with one or more of the already analysed genes will be shared. Comparing the intron rich *DapMhc1 *and *PdcMhc1 *genes with the mosquito and *Drosophila Mhc1 *genes, it is apparent that intron loss is a major determinant of arthropod *Mhc1 *gene evolution. Loss of intron events have also been found for many other arthropod genes [33]. However, as long as data from further arthropod species is missing, it cannot be excluded that some of the introns in the tail region, that are not shared between the analyzed arthropods, have been gained during evolution. Very recently, an analysis of eleven *Drosophila *genomes showed, that a small number of introns have been gained in these species [43]. The ancient *Mhc1 *gene is expected to contain all intron positions that appear in at least one of the analysed *Mhc1 *genes. Analysis of *Mhc1 *genes of further species might add additional intron positions especially in the tail region. The exon lengths of the ancient *Mhc1 *gene are between 30 and 210 bp in the motor domain and up to 480 bp in the tail region. These short exons (compared to e.g. the *Drosophila Mhc1 *gene) resemble exon lengths in vertebrates and further comparative analysis with vertebrate muscle myosin heavy chain genes will reveal the gene structure of the ancient Metazoa gene.

In addition to the *Mhc1 *gene, *Aedes aegypti *encodes a further muscle myosin heavy chain gene, named *Mhc3 *that encodes only one variant of each of the alternatively spliced exons of the *Mhc1 *gene. The presence of this gene is not an artefact from sequencing or the assembly process. Both genes, *Mhc1 *and *Mhc3*, are very different at the DNA level, and both are confirmed by several EST clones, although the translated exons show high identities. That also means, that the *Mhc3 *gene, that does not encode any alternatively spliced exons, is expressed during the life cycle of *Aedes aegypti*. However, there is not enough data that shows that the *Mhc3 *gene is expressed in a biological important (e.g. muscle-specific) manner. Note that the combination of alternatively spliced exons does not correspond to any of the tissue-specific combinations found in *Drosophila *[18]. The *Culex pipiens quinquefasciatus *genome contains another two muscle myosin heavy chain genes in addition to the *Mhc1 *gene, named *Mhc3 *and *Mhc4*, that, similarly to *AeaMhc3*, encode only one variant of most of the alternatively spliced exons of the *Mhc1 *gene. In one case, the intron between the presumed variant of the alternatively spliced exons and the following constitutive exon disappeared. Unfortunately, there is not enough EST data available for *Culex pipiens quinquefasciatus *to support any of the myosin heavy chain genes. *AeaMhc3*, *CpqMhc3*, and *CpqMhc4 *retained the same variants of the alternative exons of the corresponding *Mhc1 *genes. The presence of these further muscle myosin heavy chain genes is very surprising because the number of alternatively spliced exons in the *Mhc1 *genes already allows for the transcription of several hundred different muscle myosin isoforms. How could it happen that the genomes of *Aedes aegypti *and *Culex pipiens quinquefasciatus *encode such genes? According to the phylogenetic tree of the myosin heavy chain genes, the *Mhc3 *and *Mhc4 *genes obviously appeared in the common ancester of *Aedes *and *Culex *after the divergence from *Anopheles gambiae*. In addition, there is no evidence for a (partial) second muscle myosin heavy chain gene in the *Anopheles gambiae *genome. Also, the carboxy-terminal ends of *AeaMhc3 *and *CpqMhc4*, that are 3' elongations of the last constitutive exon, do not exist in the *AeaMhc1 *and *CpqMhc1 *genes but have an identical counterpart in the *AngMhc1 *gene that is also supported by several EST clones. It is unlikely that these three organisms have developed such a carboxy-terminal end of the myosin gene independently from each other. Instead, it is more probable that the ancient *AeaMhc1 *and *CpqMhc1 *genes have lost this specific carboxy-terminus after incorporation of the *Mhc3 *and *Mhc4 *genes into the genome. This would mean that this carboxy-terminus is only used in the specific combination of alternatively spliced exons as found in the *AeaMhc3 *and *CpqMhc4 *genes. Whether this is also true for the *AngMhc1 *gene has to be verified. Based on their identity in sequence and gene structure it is most probable that *CpqMhc3 *has been derived by gene duplication of *CpqMhc4 *or *CpqMhc4 *is a duplication of *CpqMhc3*.

**Figure 7:**
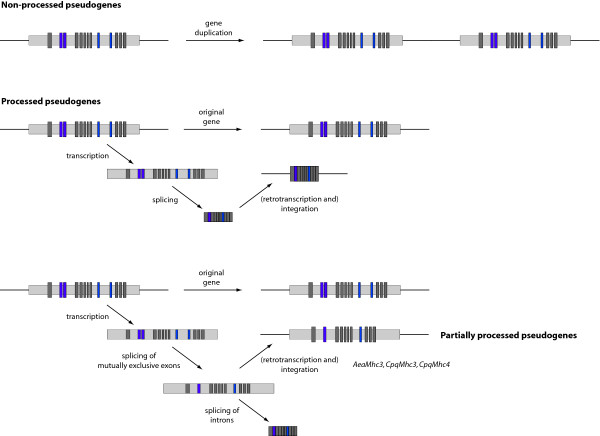
**Model for the process of alternative splicing**. The model describes the three different origins of pseudogenes. Non-processed pseudogenes are often found adjacent to their paralogous functional gene and retain the same exon-intron structure. Processed pseudogenes are marked by the absence of both 5' promotor sequence and introns, the presence of flanking direct repeats, and are randomly integrated into the genome. In the case of the arthropod *Mhc *genes, these get in the first step transcribed. In a second step, the alternative exons get spliced resulting in a certain combination of alternative exons and retaining the exon-intron structure. In the case of *AeaMhc3*, *CpqMhc3*, and *CpqMhc4*, these transcripts have been integrated into the genome. Normally in a third step, the introns get spliced revealing the final mRNA ready for translation. Dark grey bars represent constitutive and coloured bars alternatively spliced exons. Light grey bars represent non-coding sequence.

There are two possibilities as to how the *Mhc3 *and *Mhc4 *genes could have appeared in the common ancestor of *Aedes *and *Culex*. The genes have either been derived from a duplication of the *Mhc1 *gene as part of a single gene or chromosomal region duplication event. Or, a partially spliced transcript of *Mhc1 *has been reincorporated into the genome (Figure 7). If the *Mhc3 *and *Mhc4 *genes had been derived from duplication, then all variants except one of the alternative exons of only one of the (then) two *Mhc *genes had to be lost in addition to the loss of both terminal exons in *Mhc3*. Given the number of possible transcripts of the *Mhc1 *gene and the possibility to duplicate alternative exons, it is very unlikely that there would be a need for a second gene with the same set of alternative exons. If it were advantageous to keep two almost identical genes, it would be very unlikely that only one of the genes has lost all except one of its alternative exons. In addition, there must have been a very strong evolutionary pressure to keep exactly this special combination of alternative exons. The second possibility would mean that in the first step during the splicing process all alternatively spliced exons, which are not needed, are removed leaving introns between the remaining alternatively spliced and constitutive exons (Figure 7). In the second step, all introns are spliced to yield the mRNA for translation. In the case of the *Mhc3 *and *Mhc4 *genes, the transcript containing one combination of alternative exons but all introns would have been integrated into the genome, probably after retrotranscription. How should this type of genes be called? At least the *AeaMhc3 *gene is completely transcribed, and also *CpqMhc3 *and *CpqMhc4 *do not contain any premature stop codons or frameshift mutations. However, compared to the corresponding *Mhc1 *genes they retained only one variant exon of each of the alternative exons. Thus, they do not belong to the non-processed pseudogenes. We would rather regard them as a new type of partially processed pseudogenes.

## Conclusion

25 arthropod muscle myosin heavy chain genes have been identified and analysed. Compared to the well-studied gene of *Drosophila melanogaster *other arthropod genes might contain up to four additional alternatively spliced exons encoding part of the motor domain. This considerably extends the possibilities of other arthropod species to fine-tune myosin and thus muscle characteristics. An ancient arthropod muscle myosin heavy chain gene has been reconstructed whose gene structure can best be explained if introns are lost and not gained during evolution of this gene. *Aedes aegypti *and *Culex pipiens quinquefasciatus *even encode further muscle myosin heavy chain genes that, however, have lost all except one variant of the alternatively spliced exons. These genes most probably entered the genome by reincorporating a certain processed transcript and not via a gene or genomic region duplication event. If the gene has been derived from a processed transcript then splicing of alternative exons must involve a first step, in which all other variants are spliced out leaving intronic sequence around the variant of choice. In a second step, all introns are spliced.

## Methods

### Identification and annotation of the arthropod muscle myosin heavy chains

The genes for *Aea*, *Ang*, *Am*, *Bm*, *Cpq*, *Dm*, *Drp*, *Dp*, *Dse*, *Dss*, *Dy*, *Dw*, *Pdc*, and *Tic Mhc1 *and *Mhc3 *have been obtained by TBLASTN searches against the insects section of the NCBI wgs database (Table 1)[44]. The genes for the *Da*, *Der*, *Dg*, *Dmo*, and *Dv Mhc1 *have been obtained using the BLAT alignment tool [45] against the UCSC Genome Browser database [46,47]. The *DhMhc1 *sequence was derived from the NCBI nonredundant database. The *DapMhc1 *sequence has been obtained by a TBLASTN search against the 9× assembly of the *Daphnia pulex *genome provided by the DOE Joint Genome Institute [48] and the *Daphnia *Genomics Consortium [49]. The *NavMhc1 *gene was derived from version 1.0 of the *Nasonia vitripennis *assembly provided by the Human Genome Sequencing Center at Baylor College of Medicine [50]. The exons of the genes were predicted by manual inspection of the nucleotide sequences. For the correct prediction of the transcriptional start and the 3' terminal exons, the analysis of cDNA and EST data, that has been obtained from the EST section of NCBI's nucleotide database, was necessary. In particular, the following data has been obtained: For *TicMhc1*, only a small amount of EST data is available, confirming the prediction of exon2. There is not enough data to exclude a further untranslated 5' exon, as well as further C-terminal exons. For *AngMhc1*, several EST and cDNA clones support exon1 and the different C-termini. The C-termini of *AeaMhc1 *are also supported by several EST clones (e.g. GenBank ID DV384821). Exon1 of *AeaMhc3 *is supported by EST data. Exon1 of *AeaMhc3 *has been used for the identification of exon1 of *AeaMhc1*, as there is no direct evidence by EST data. Surprisingly, it is found 26,432 bp before the translation start codon ATG. For *AmMhc1*, the N-terminus is not supported by EST or cDNA data. Therefore it is not clear whether there might be an additional 5' untranslated exon. The C-termini are supported by several EST and cDNA clones (e.g. GenBank ID CK629939). The C-terminus of *DapMhc1 *is supported by EST data (e.g. GenBank ID BJ927473), while there is no EST data for the N-terminus. For *BmMhc1*, exon2 is supported by EST data. However, the corresponding EST clones are not long enough to exclude a further 5' untranslated exon. Both C-termini of *BmMhc1 *are supported by EST clones (e.g. GenBank ID BP179837). The genomic DNA of the *BmMhc1 *gene contains a gap in the coiled-coil tail region. The missing amino acid sequence has been derived from EST data. However, the exon/intron structure in the corresponding region remains unresolved.

### Analysis of the relationship of the alternatively spliced exons

All alternatively spliced exons have been aligned manually. Some kind of relationship is already obvious from these sequence alignments. To get a more quantitative description, sequence identity matrices have been calculated for each set of aligned exons. Subsequently, sets of homologous exons from all *Mhc1 *genes have been clustered by sequence similarity. We have visualized the results in graphs that have to be read in columns. The highest identity between an exon listed on top and any variant of a certain Mhc1 protein listed on the left side has been set to 1 (red colour) while the differences between the values of the lower identity exons and the value of the highest identity have been plotted for the other combinations of exons. Thus, in every column the highest identity of the named exon to one of the variants of the other Mhc1 proteins is visualized.

### Building trees

The phylogenetic tree was generated using neighbour joining and the Bootstrap (1,000 replicates) method as implemented in ClustalW (standard settings) [51] and drawn by using TreeView [52]. The sequence of *DapMhc1 *has been used as outgroup.

## List of abbreviations

Mhc, myosin heavy chain; for abbreviations of species names see Table 1.

## Authors' contributions

F.O. performed data analysis. M.K. assembled all sequences, performed data analysis and wrote the manuscript. Both authors read and approved the manuscript.

## Supplementary Material

Additional file 1 **Mhc1 sequence alignment**. The file contains the aligned arthropod Mhc1 protein sequences. Also included are all variants of the alternatively spliced exons. Click here for file

Additional file 2 **Sequence alignment and analysis of the alternatively spliced exons**. The file contains the aligned alternative exons of the arthropod Mhc1 protein sequences. Also included are the graphical representations of the sequence identities. Click here for file
