# Supplementary material for: Comparative genomic analysis of the arthropod muscle myosin heavy chain genes allows ancestral gene reconstruction and reveals a new type of 'partially' processed pseudogene
Source: BMC Mol Biol. 2008 Feb 6;9:21. doi: 10.1186/1471-2199-9-21 (PMC2257972; doi:10.1186/1471-2199-9-21)
Supplement: Additional file 2 — Sequence alignment and analysis of the alternatively spliced exons. The file contains the aligned alternative exons of the arthropod Mhc1 protein sequences. Also included are the graphical representations of the sequence identities. [file 1471-2199-9-21-S2.pdf]

Additional File 2

Figures 1-9 show the protein sequence alignments of the alternative exons. The upper sequences, termed Mhc1, Mhc3, and Mhc4, respectively, represent either the variant “a” exons (e.g. Figure 1 or Figure 3) or the corresponding parts of constitutive exons (e.g. Figure 2).

Figure 2 and Figure 6A: Most genes do not have introns at the positions corresponding to the alternatively spliced *Dap*, *Tic*, *Pdc,* and *Bm* exons. Because these exons span the variable loop-1 (Figure 2) and loop2 (Figure 6A) regions their start and end positions are not conserved. In Figure 6A, the variant “b” sequences of *Bm*, *Dap*, *Pdc*, and *Tic* have been directly aligned with their corresponding variant “a” sequences.

Figure 9: Alignment of the potential carboxy-termini of the *Mhc* genes. For better understanding a gap (-) has been introduced between the last constitutive exon and the following alternative exons. In the case of *AeaMhc3*, *CpqMhc3*, *CpqMhc4*, and *AngExon15* the carboxy-termini are elongations of the last constitutive exon and not separated exons.

The alignments of the sections of the *Mhc1* genes of Figure 1 (main manuscript) showing the relationship between the exon-intron structures of the regions containing the alternatively spliced exons have been taken from Figure 2 (main manuscript) for better comparison. Continuous lines connect variants that are almost identical and thus expected to be derived from a common ancestor. Bold lines connecting alternative exons in regions containing multiple variants per *Mhc1* gene highlight particularly conserved exons in these sets. Dotted lines represent putative connections between certain variants although their identity is not very strong on the protein level.

For the construction of the figures showing the comparison of the sequence identity between each exon and all variant exons of every other Mhc1 proteins see Materials and Methods in the main manuscript. For interpretation, the figures have to be read in columns. The highest identity between an exon listed on top and any variant of a certain Mhc1 protein listed on the left side has been set to 1 (red color) while the differences of the values of the lower identity exons to the value of the highest identity have been plotted for the other combinations of exons. Thus, in every column the highest identity of the named exon to one of the variants of the other Mhc1 proteins is visualized.

Figure 1

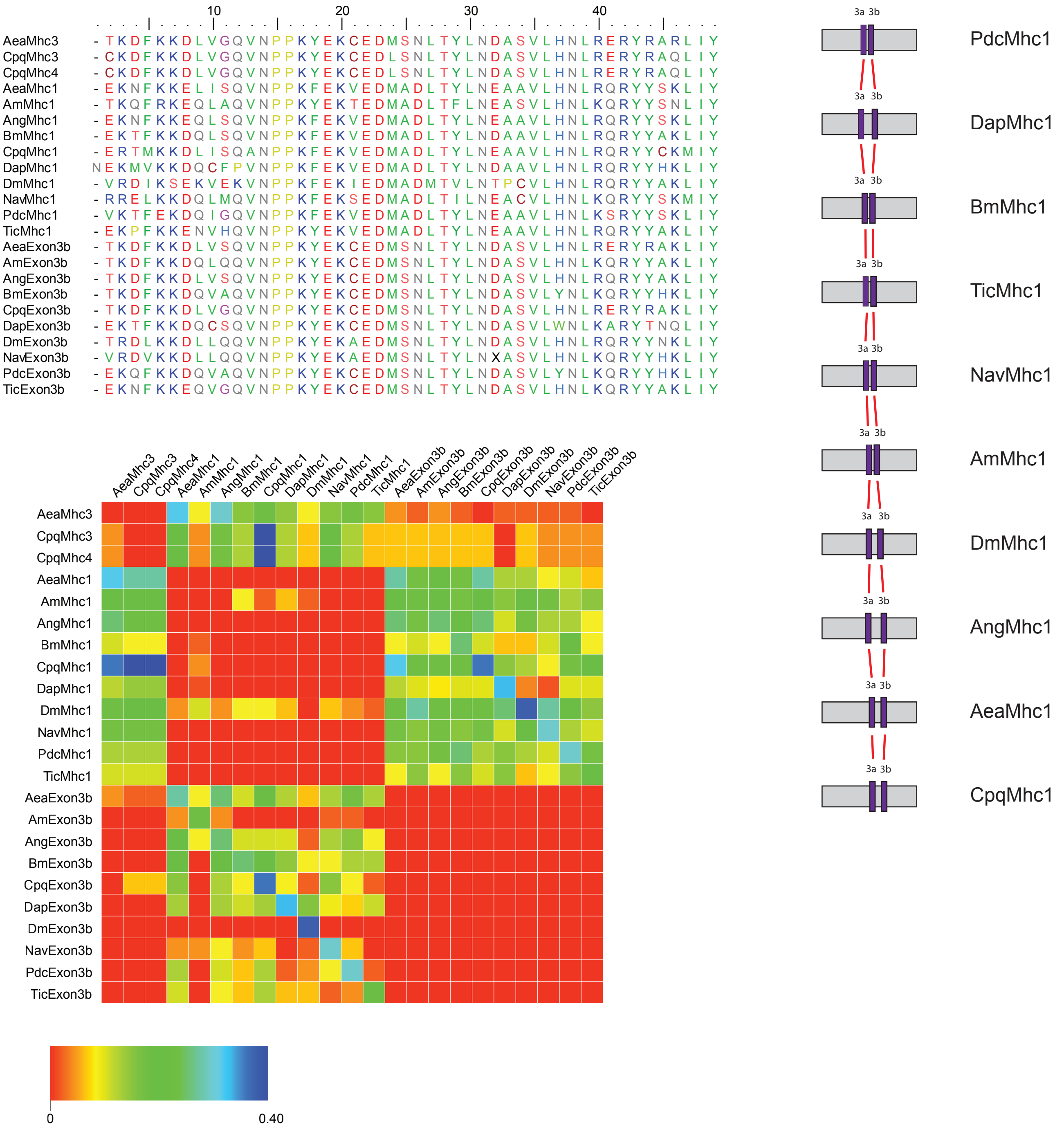

10 20 30 40
AeaMhc3 - TKDFKKDLVGQVNPPKYEKCEDMSNLTYLNDASVLHNLRERYRARLIY
CpqMhc3 - CKDFKKDLVGQVNPPKYEKCEDLSNLTYLNDASVLHNLRERYRAQLIY
CpqMhc4 - CKDFKKDLVGQVNPPKYEKCEDLSNLTYLNDASVLHNLRERYRAQLIY
AeaMhc1 - EKNFKKELISQVNPPKFEKVEDMADLTYLNEAAVLHNLRQRYYSKLIY
AmMhc1 - TKQFRKEQLAQVNPPKYEKTEDMADLTFLNEASVLHNLKQRYYSNLIY
AngMhc1 - EKNFKKEQLSQVNPPKFEKVEDMADLTYLNEAAVLHNLRQRYYSKLIY
BmMhc1 - EKTFKKDQLSQVNPPKFEKVEDMADLTYLNDAAVLHNLRQRYYAKLIY
CpqMhc1 - ERTMKKDLISQANPPKFEKVEDMADLTYLNEAAVLHNLRQRYYCKMIY
DapMhc1 NEKMVKKDQCFPVNPPKFEKVEDMADLTYLNDAAVLHNLRQRYYHKLIY
DmMhc1 - VRDIKSEKVEKVNPPKFEKIEDMADMTVLNTPCVLHNLRQRYYAKLIY
NavMhc1 - RRELKKDQLMQVNPPKFEKSEDMADLTILNEACVLHNLKQRYYSKMIY
PdcMhc1 - VKTFEKDQIGQVNPPKFEKVEDMADLTYLNEAAVLHNLKSRYYSKLIY
TicMhc1 - EKPFKKENVHQVNPPKYEKVEDMADLTYLNEAAVLHNLRQRYYAKLIY
AeaExon3b - TKDFKKDLVSQVNPPKYEKCEDMSNLTYLNDASVLHNLRERYRAKLIY
AmExon3b - TKDFKKDQLQQVNPPKYEKCEDMSNLTYLNDASVLHNLKQRYYAKLIY
AngExon3b - TKDFKKDLVSQVNPPKYEKCEDMSNLTYLNDASVLHNLRQRYYAKLIY
BmExon3b - TKDFKKDQVAQVNPPKYEKCEDMSNLTYLNDASVLYNLKQRYYHKLIY
CpqExon3b - TKDFKKDLVGQVNPPKYEKCEDMSNLTYLNDASVLHNLRERYRAKLIY
DapExon3b - EKTFKKDQCSQVNPPKYEKCEDMSNLTYLNDASVLWNLKARYTNQLIY
DmExon3b - TRDLKKDLLQQVNPPKYEKAEDMSNLTYLNDASVLHNLRQRYYNKLIY
NavExon3b - VRDVKKDLLQQVNPPKYEKAEDMSNLTYLNXASVLHNLKQRYYHKLIY
PdcExon3b - EKQFKKDQVAQVNPPKYEKCEDMSNLTYLNDASVLYNLKQRYYHKLIY
TicExon3b - EKNFKKEQVGQVNPPKYEKCEDMSNLTYLNDASVLHNLKQRYYAKLIY
AeaMhc3
CpqMhc3
CpqMhc4
AeaMhc1
AmMhc1
AngMhc1
BmMhc1
CpqMhc1
DapMhc1
DmMhc1
NavMhc1
PdcMhc1
TicMhc1
AeaExon3b
AmExon3b
AngExon3b
BmExon3b
CpqExon3b
DapExon3b
DmExon3b
NavExon3b
PdcExon3b
TicExon3b
0
0.40
3a 3b
PdcMhc1
DapMhc1
BmMhc1
TicMhc1
NavMhc1
AmMhc1
DmMhc1
AngMhc1
AeaMhc1
CpqMhc1

Figure 2

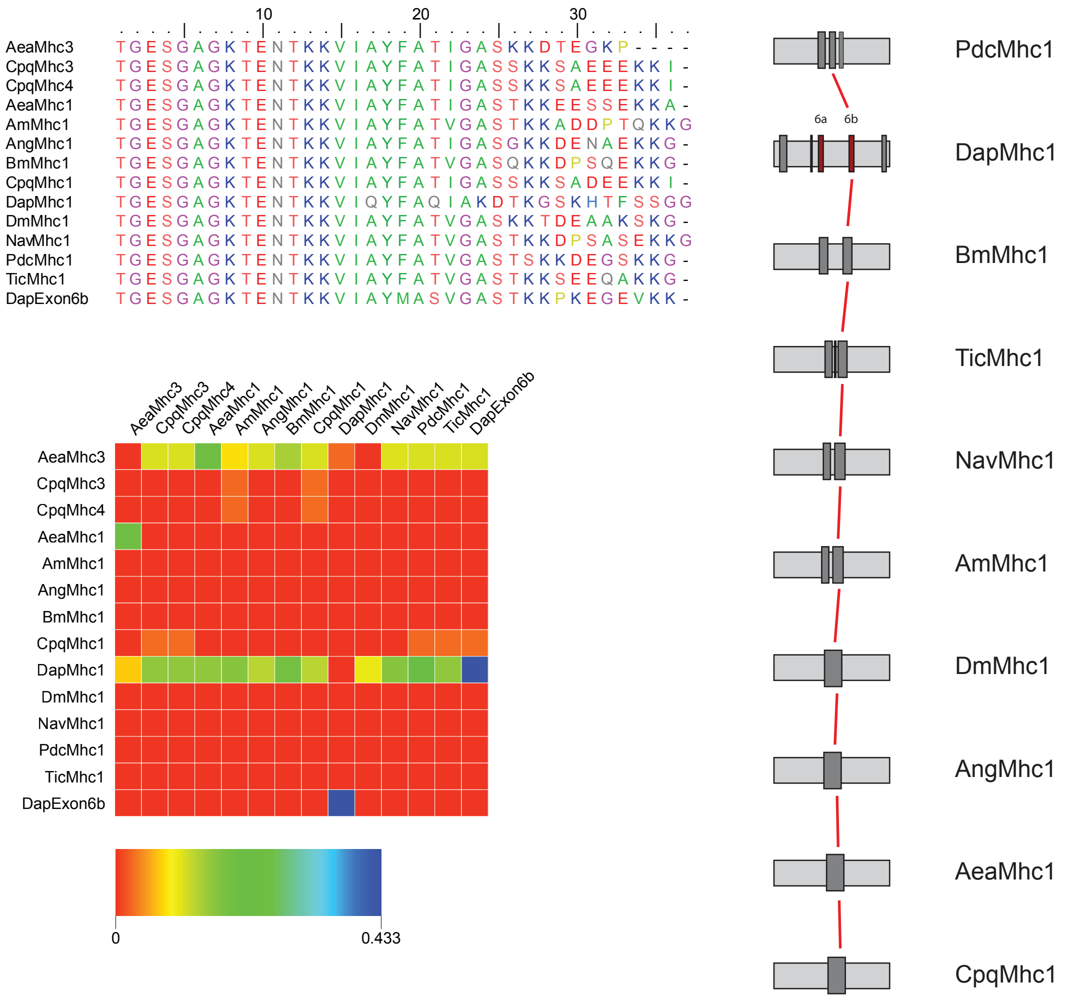

10
20
30
AeaMhc3 TGESGAGKTENTKKVIAYFATIGASKKDTEGKP----
CpqMhc3 TGESGAGKTENTKKVIAYFATIGASSKKSAEEEKKI-
CpqMhc4 TGESGAGKTENTKKVIAYFATIGASSKKSAEEEKKI-
AeaMhc1 TGESGAGKTENTKKVIAYFATIGASTKKEESSEKKA-
AmMhc1 TGESGAGKTENTKKVIAYFATVGASTKKADDPTQKKG
AngMhc1 TGESGAGKTENTKKVIAYFATIGASGKKDENAEKKG-
BmMhc1 TGESGAGKTENTKKVIAYFATVGASQKKDPSQEKKG-
CpqMhc1 TGESGAGKTENTKKVIAYFATIGASSKKSADEEKKI-
DapMhc1 TGESGAGKTENTKKVIQYFAQIAKDTKGSKHTFSSGG
DmMhc1 TGESGAGKTENTKKVIAYFATVGASKKTDEAAKSKG-
NavMhc1 TGESGAGKTENTKKVIAYFATVGASTKKDPSASEKKG
PdcMhc1 TGESGAGKTENTKKVIAYFATVGASTSKKDEGSKKG-
TicMhc1 TGESGAGKTENTKKVIAYFATVGASTKKSEEQAKKG-
DapExon6b TGESGAGKTENTKKVIAYMASVGASTKKPKEGEVKK-
AeaMhc3
CpqMhc3
CpqMhc4
AeaMhc1
AmMhc1
AngMhc1
BmMhc1
CpqMhc1
DapMhc1
DmMhc1
NavMhc1
PdcMhc1
TicMhc1
DapExon6b
0
0.433
PdcMhc1
6a
6b
DapMhc1
BmMhc1
TicMhc1
NavMhc1
AmMhc1
DmMhc1
AngMhc1
AeaMhc1
CpqMhc1

Figure 3

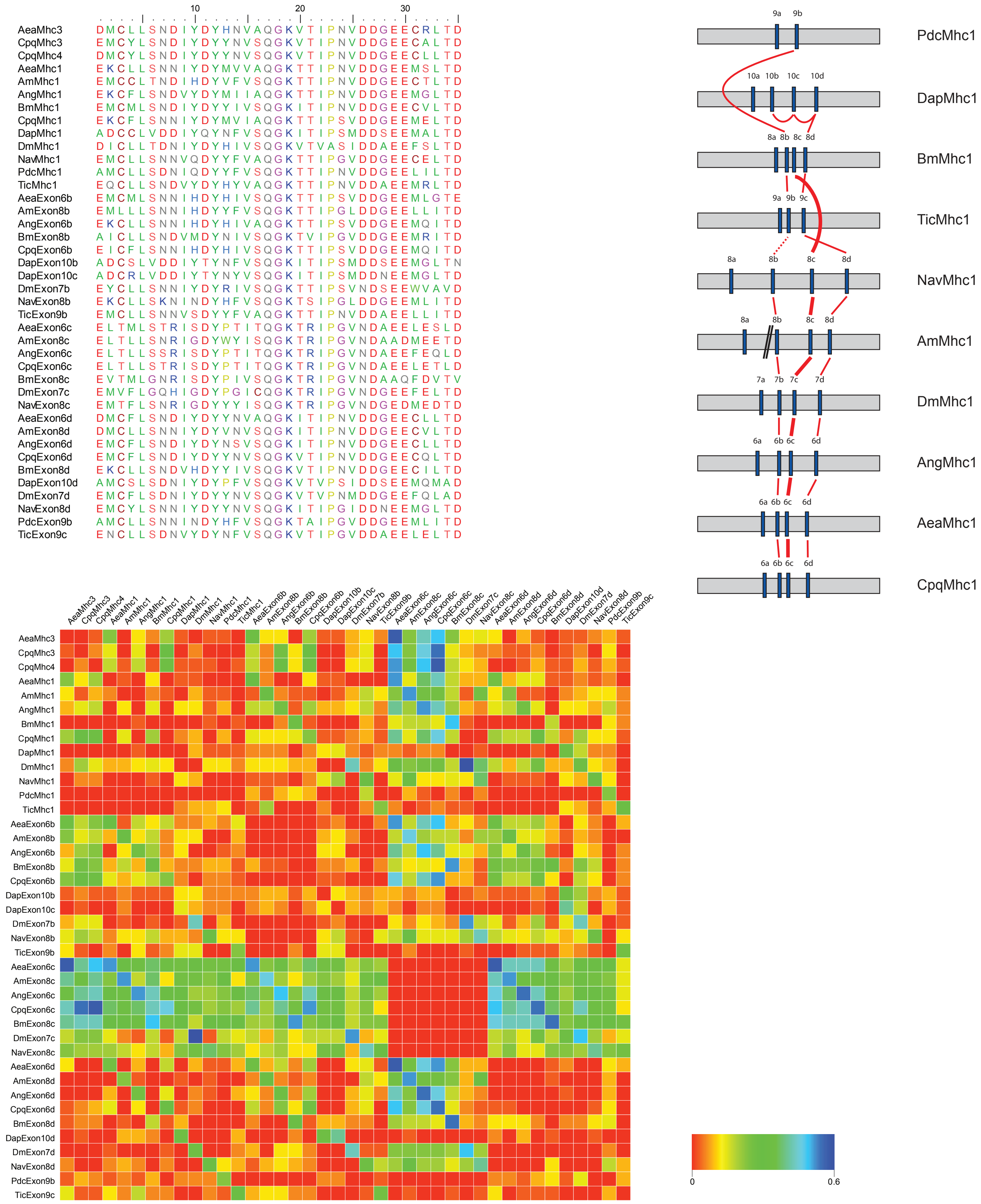

10
20
30
AeaMhc3 DMCLLSNDIYDYHNVAQGKVTIPNVDDGEECRLTD
CpqMhc3 EMCYLSNDIYDYYNVSQGKVTIPNVDDGEECALTD
CpqMhc4 DMCYLSNDIYDYYNVSQGKVTIPNVDDGEECLLTD
AeaMhc1 EKCLLSNNIYDYMVVAQGKTTIPNVDDGEEMSLTD
AmMhc1 EMCCLTNDIHDYVFVSQGKTTIPNVDDGEECTLTD
AngMhc1 EKCFLSNDVYDYMIIAQGKTTIPNVDDGEEMGLTD
BmMhc1 EMCMLSNDIYDYYIVSQGKITIPNVDDGEECVLTD
CpqMhc1 EKCFLSNNIYDYMVIAQGKTTIPSVDDGEEMELTD
DapMhc1 ADCCLVDDIYQYNFVSQGKITIPSMDDSEEMALTD
DmMhc1 DICLLTDNIYDYHIVSQGKVTVASIDDAEEFSLTD
NavMhc1 EMCLLSNNVQDYYFVAQGKTTIPGVDDGEECELTD
PdcMhc1 AMCLLSDNIQDYYFVSQGKTTIPNVDDGEELILTD
TicMhc1 EQCLLSNDVYDYHYVAQGKTTIPNVDDAEEMRLTD
AeaExon6b EMCMLSNNIHDYHIVSQGKTTIPSVDDGEEMLGTE
AmExon8b EMLLLSNNIHDYYFVSQGKTTIPGLDDGEELLITD
AngExon6b EKCLLSNNIHDYHIVAQGKTTIPSVDDGEEMQITD
BmExon8b AICLLSNDVMDYNIVSQGKTVIPGVDDGEEMRITD
CpqExon6b EICFLSNNIHDYHIVSQGKTTIPSVDDGEEMQITD
DapExon10b ADCSLVDDIYTYNFVSQGKITIPSMDDSEEMGLTN
DapExon10c ADCRLVDDIYTYNFVSQGKITIPSMDDNEEMGLTD
DmExon7b EYCLLSNNIYDYRIVSQGKTTIPSVNDSEEWVAVD
NavExon8b EKCLLSKNINDYHFVSQGKTSIPGLDDGEEMLITD
TicExon9b EMCLLSNNVSDYYFVAQGKTTIPNVDDAEELLITD
AeaExon6c ELTMLSTRISDYPTITQGKTRIPGVNDAEELESLD
AmExon8c ELTLLSNRIGDYWYISQGKTRIPGVNDAADMEETD
AngExon6c ELTLLSSRISDYPTITQGKTRIPGVNDAEEFEQLD
CpqExon6c ELTLLSTRISDYPTITQGKTRIPGVNDAEELETLD
BmExon8c EVTMLGNRISDYPIVSQGKTRIPGVNDAAQFDVTV
DmExon7c EMVFLGQHIGDYPGICQGKTRIPGVNDGEEFELTD
NavExon8c EMTFLSNRIGDYYYISQGKTRIPGVNDGEDMEDTD
AeaExon6d DMCFLSNDIYDYYNVAQGKITIPNVDDGEECLLTD
AmExon8d DMCLLSNNIYDYVNVSQGKITIPNVDDGEECVLTD
AngExon6d EMCFLSNDIYDYNSVSQGKITIPNVDDGEECLLTD
CpqExon6d EMCFLSNDIYDYYNVSQGKVTIPNVDDGEECQLTD
BmExon8d EKCLLSNDVHDYYIVSQGKVTIPNVDDGEECILTD
DapExon10d AMCSLSDNIYDYPFVSQGKVTVPSIDDSEEMQMAD
DmExon7d EMCFLSDNIYDYYNVSQGKVTVPNMDDGEEFQLAD
NavExon8d EMCYLSNNIYDYYNVSQGKITIPGIDDNEEMGLTD
PdcExon9b AMCLLSNNINDYHFVSQGKTAIPGVDDGEEMLITD
TicExon9c ENCLLSDNVYDYNFVSQGKVTIPGVDDAEELELTD
PdcMhc1
9a 9b
DapMhc1
10a 10b 10c 10d
BmMhc1
8a 8b 8c 8d
TicMhc1
9a 9b 9c
NavMhc1
8a 8b 8c 8d
AmMhc1
8a 8b 8c 8d
DmMhc1
7a 7b 7c 7d
AngMhc1
6a 6b 6c 6d
AeaMhc1
6a 6b 6c 6d
CpqMhc1
6a 6b 6c 6d
0
0.6

Figure 4

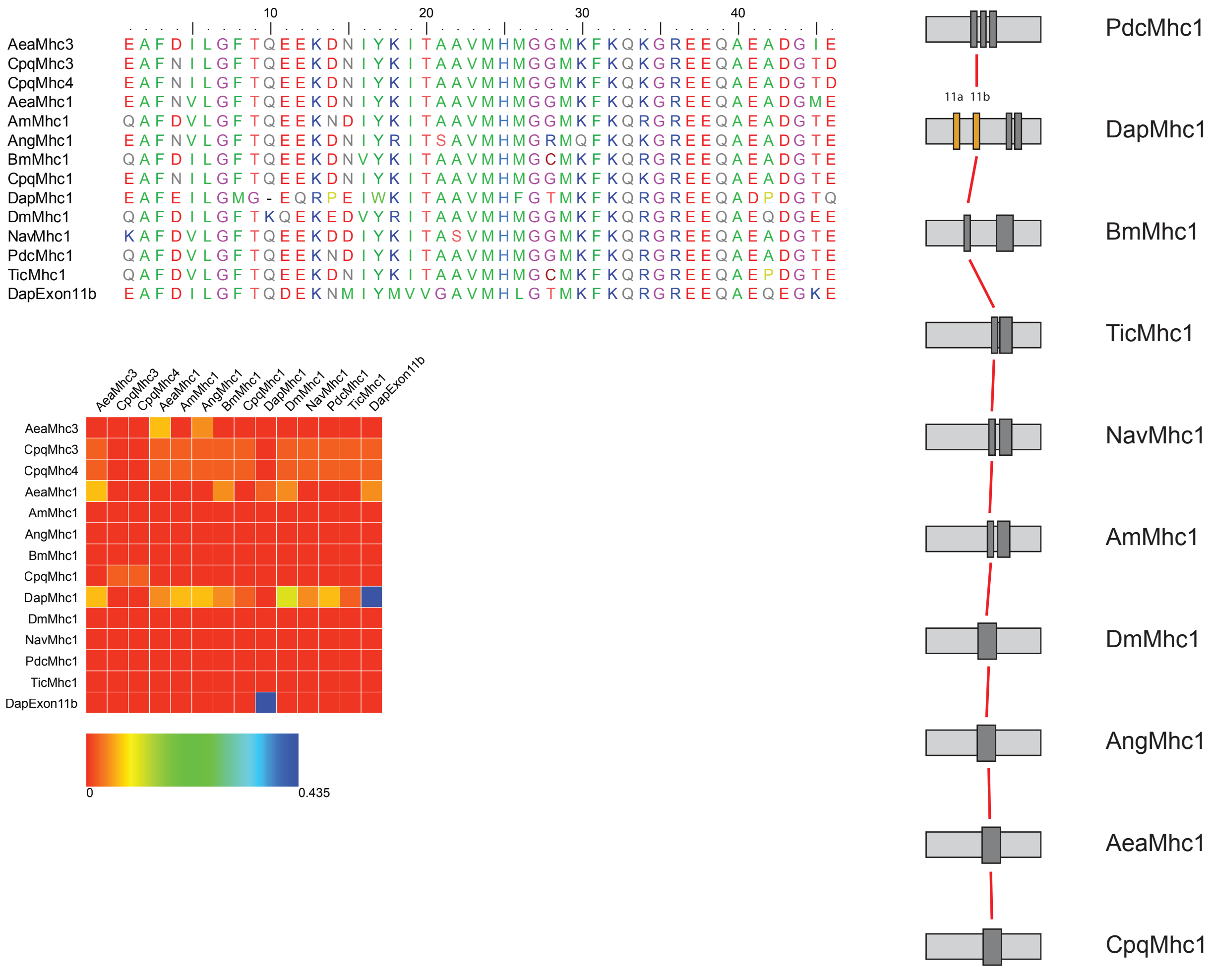

10
20
30
40
AeaMhc3 EAFDILGFTQEEKDNIYKITAAVMHMGGMKFKQKGREEQAEADGIE
CpqMhc3 EAFNILGFTQEEKDNIYKITAAVMHMGGMKFKQKGREEQAEADGTD
CpqMhc4 EAFNILGFTQEEKDNIYKITAAVMHMGGMKFKQKGREEQAEADGTD
AeaMhc1 EAFNVLGFTQEEKDNIYKITAAVMHMGGMKFKQKGREEQAEADGME
AmMhc1 QAFDVLGFTQEEKNDIYKITAAVMHMGGMKFKQRGREEQAEADGTE
AngMhc1 EAFNVLGFTQEEKDNIYRITSAVMHMGRMQFKQKGREEQAEADGTE
BmMhc1 QAFDILGFTQEEKDNVYKITAAVMHMGCMKFKQRGREEQAEADGTE
CpqMhc1 EAFNILGFTQEEKDNIYKITAAVMHMGGMKFKQKGREEQAEADGTE
DapMhc1 EAFEILGMG-EQRPEIWKITAAVMHFGTMKFKQRGREEQADPDGTQ
DmMhc1 QAFDILGFTKQEKEDVYRITAAVMHMGGMKFKQRGREEQAEQDGEE
NavMhc1 KAFDVLGFTQEEKDDIYKITASVMHMGGMKFKQRGREEQAEADGTE
PdcMhc1 QAFDVLGFTQEEKNDIYKITAAVMHMGGMKFKQRGREEQAEADGTE
TicMhc1 QAFDVLGFTQEEKDNIYKITAAVMHMGCMKFKQRGREEQAEPDGTE
DapExon11b EAFDILGFTQDEKNMIYMVVGAVMHLGTMKFKQRGREEQAEQEGKE
AeaMhc3
CpqMhc3
CpqMhc4
AeaMhc1
AmMhc1
AngMhc1
BmMhc1
CpqMhc1
DapMhc1
DmMhc1
NavMhc1
PdcMhc1
TicMhc1
DapExon11b
0
0.435
PdcMhc1
11a 11b
DapMhc1
BmMhc1
TicMhc1
NavMhc1
AmMhc1
DmMhc1
AngMhc1
AeaMhc1
CpqMhc1

Figure 5

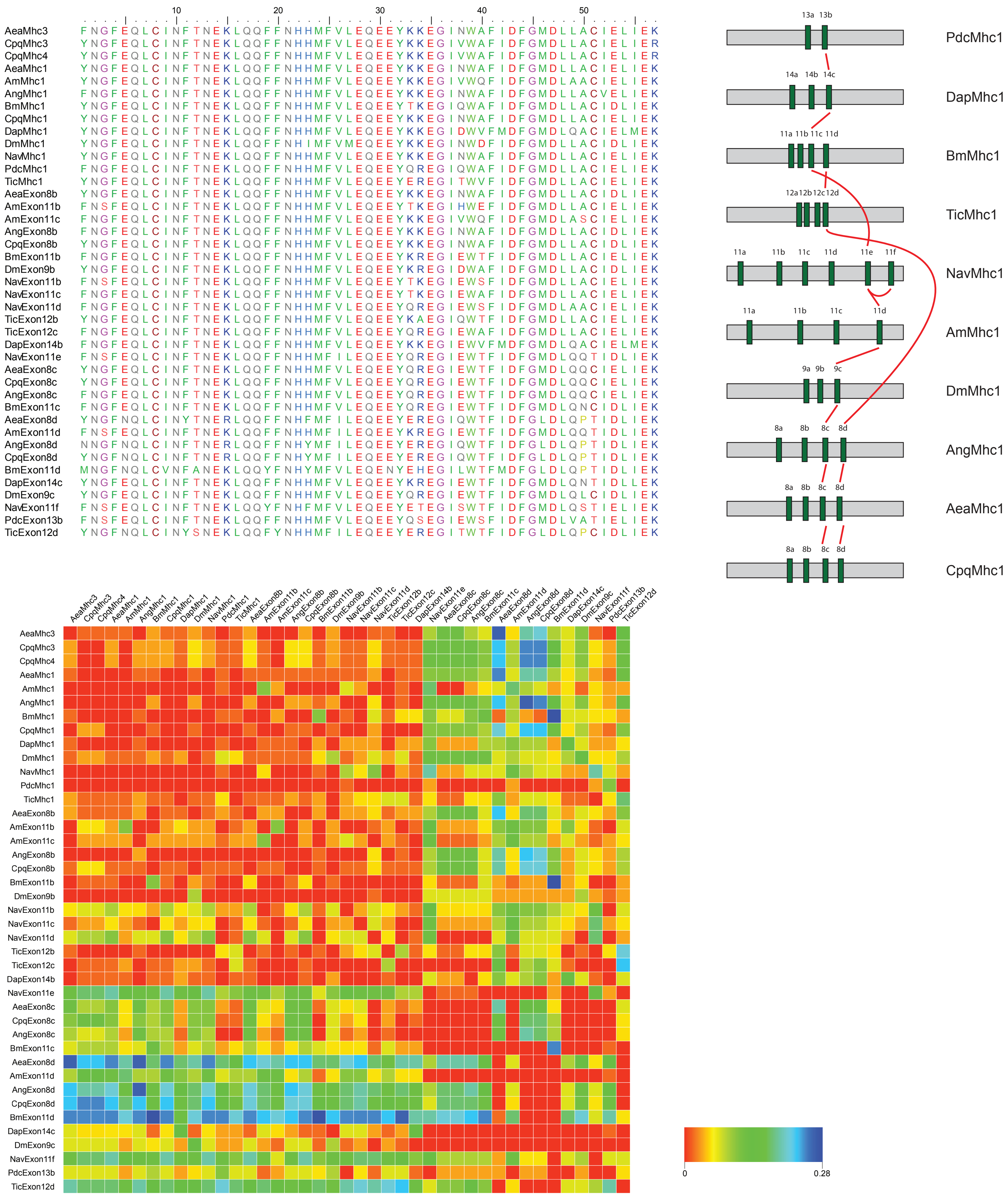

10
20
30
40
50
AeaMhc3 FNGFEQLCINFTNEKLQQFFNHHMFVLEQEEYKKEGINWAFIDFGMDLLACIELIEK
CpqMhc3 YNGFEQLCINFTNEKLQQFFNHHMFVLEQEEYKKEGIVWAFIDFGMDLLACIELIER
CpqMhc4 YNGFEQLCINFTNEKLQQFFNHHMFVLEQEEYKKEGIVWAFIDFGMDLLACIELIER
AeaMhc1 YNGFEQLCINFTNEKLQQFFNHHMFVLEQEEYKKEGINWAFIDFGMDLLACIELIEK
AmMhc1 YNGFEQLCINFTNEKLQQFFNHHMFVLEQEEYKKEGIVWQFIDFGMDLAACIELIEK
AngMhc1 FNGFEQLCINFTNEKLQQFFNHHMFVLEQEEYKKEGINWAFIDFGMDLLACVELIEK
BmMhc1 YNGFEQLCINFTNEKLQQFFNHHMFVLEQEEYTKEGIQWAFIDFGMDLLACIDLIEK
CpqMhc1 YNGFEQLCINFTNEKLQQFFNHHMFVLEQEEYKKEGINWAFIDFGMDLLACIELIEK
DapMhc1 YNGFEQLCINFTNEKLQQFFNHHMFVLEQEEYKKEGIDWVFMDFGMDLQACIELMEK
DmMhc1 YNGFEQLCINFTNEKLQQFFNHIMFVMEQEEYKKEGINWDFIDFGMDLLACIDLIEK
NavMhc1 YNGFEQLCINFTNEKLQQFFNHHMFVLEQEEYKKEGINWAFIDFGMDLLACIELIEK
PdcMhc1 FNGFEQLCINFTNEKLQQFFNHHMFVLEQEEYQREGIQWAFIDFGMDLLACIELIEK
TicMhc1 YNGFEQLCINFTNEKLQQFFNHHMFVLEQEEYEREGITWVFIDFGMDLLACIELIEK
AeaExon8b YNGFEQLCINFTNEKLQQFFNHHMFVLEQEEYKKEGINWAFIDFGMDLLACIDLIEK
AmExon11b FNSFEQLCINFTNEKLQQFFNHHMFVLEQEEYTKEGIHWEFIDFGMDLLACIELIEK
AmExon11c FNGFEQLCINFTNEKLQQFFNHHMFVLEQEEYKKEGIVWQFIDFGMDLASCIELIEK
AngExon8b FNGFEQLCINFTNEKLQQFFNHHMFVLEQEEYKKEGINWAFIDFGMDLLACIDLIEK
CpqExon8b YNGFEQLCINFTNEKLQQFFNHHMFVLEQEEYKKEGINWAFIDFGMDLLACIDLIEK
BmExon11b FNGFEQLCINFTNEKLQQFFNHHMFVLEQEEYKREGIEWTFIDFGMDLLACIELIEK
DmExon9b YNGFEQLCINFTNEKLQQFFNHHMFVLEQEEYKREGIDWAFIDFGMDLLACIDLIEK
NavExon11b FNSFEQLCINFTNEKLQQFFNHHMFVLEQEEYTKEGIEWSFIDFGMDLLACIELIEK
NavExon11c FNGFEQLCINFTNEKLQQFFNHHMFVLEQEEYTKEGIEWAFIDFGMDLLACIELIEK
NavExon11d FNGFEQLCINFTNEKLQQFFNHHMFVLEQEEYQREGIEWSFIDFGMDLAACIELIEK
TicExon12b YNGFEQLCINFTNEKLQQFFNHHMFVLEQEEYKAEGIQWTFIDFGMDLLACIELIEK
TicExon12c FNGFEQLCINFTNEKLQQFFNHHMFVLEQEEYQREGIEWAFIDFGMDLLACIELIEK
DapExon14b FNGFEQLCINFTNEKLQQFFNHHMFVLEQEEYKKEGIEWVFMDFGMDLQACIELMEK
NavExon11e FNSFEQLCINFTNEKLQQFFNHHMFILEQEEYQREGIEWTFIDFGMDLQQTIDLIEK
AeaExon8c YNGFEQLCINFTNEKLQQFFNHHMFVLEQEEYQREGIEWTFIDFGMDLQQCIELIEK
CpqExon8c YNGFEQLCINFTNEKLQQFFNHHMFVLEQEEYQREGIEWTFIDFGMDLQQCIELIEK
AngExon8c FNGFEQLCINFTNEKLQQFFNHHMFVLEQEEYQREGIEWTFIDFGMDLQQCIELIEK
BmExon11c FNGFEQLCINFTNEKLQQFFNHHMFVLEQEEYQREGIEWTFIDFGMDLQNCIDLIEK
AeaExon8d YNGFNQLCINYTNERLQQFFNHFMFILEQEEYEREGIQWTFIDFGLDLQPTIDLIEK
AmExon11d FNSFEQLCINFTNEKLQQFFNHHMFILEQEEYKREGIEWTFIDFGMDLQQTIDLIEK
AngExon8d NNGFNQLCINFTNERLQQFFNHYMFILEQEEYEREGIQWTFIDFGLDLQPTIDLIEK
CpqExon8d YNGFNQLCINFTNERLQQFFNHYMFILEQEEYEREGIQWTFIDFGLDLQPTIDLIEK
BmExon11d MNGFNQLCVNFANEKLQQYFNHYMFVLEQENYEHEGILWTFMDFGLDLQPTIDLIEK
DapExon14c YNGFEQLCINFTNEKLQQFFNHHMFVLEQEEYKREGIEWTFIDFGMDLQNTIDLLEK
DmExon9c YNGFEQLCINFTNEKLQQFFNHHMFVLEQEEYQREGIEWTFIDFGMDLQLCIDLIEK
NavExon11f FNSFEQLCINFTNEKLQQYFNHFMFVLEQEEYETEGISWTFIDFGMDLQSTIELIEK
PdcExon13b FNSFEQLCINFTNEKLQQFFNHHMFVLEQEEYQSEGIEWSFIDFGMDLVATIELIEK
TicExon12d YNGFNQLCINYSNEKLQQFYNHHMFILEQEEYEREGITWTFIDFGLDLQPCIDLIEK
13a 13b
PdcMhc1
14a 14b 14c
DapMhc1
11a 11b 11c 11d
BmMhc1
12a 12b 12c 12d
TicMhc1
11a 11b 11c 11d 11e 11f
NavMhc1
11a 11b 11c 11d
AmMhc1
9a 9b 9c
DmMhc1
8a 8b 8c 8d
AngMhc1
8a 8b 8c 8d
AeaMhc1
8a 8b 8c 8d
CpqMhc1
AeaMhc3
CpqMhc3
CpqMhc4
AeaMhc1
AmMhc1
AngMhc1
BmMhc1
CpqMhc1
DapMhc1
DmMhc1
NavMhc1
PdcMhc1
TicMhc1
AeaExon8b
AmExon11b
AmExon11c
AngExon8b
CpqExon8b
BmExon11b
DmExon9b
NavExon11b
NavExon11c
NavExon11d
TicExon12b
TicExon12c
DapExon14b
NavExon11e
AeaExon8c
CpqExon8c
AngExon8c
BmExon11c
AeaExon8d
AmExon11d
AngExon8d
CpqExon8d
BmExon11d
DapExon14c
DmExon9c
NavExon11f
PdcExon13b
TicExon12d
0
0.28

Figure 6

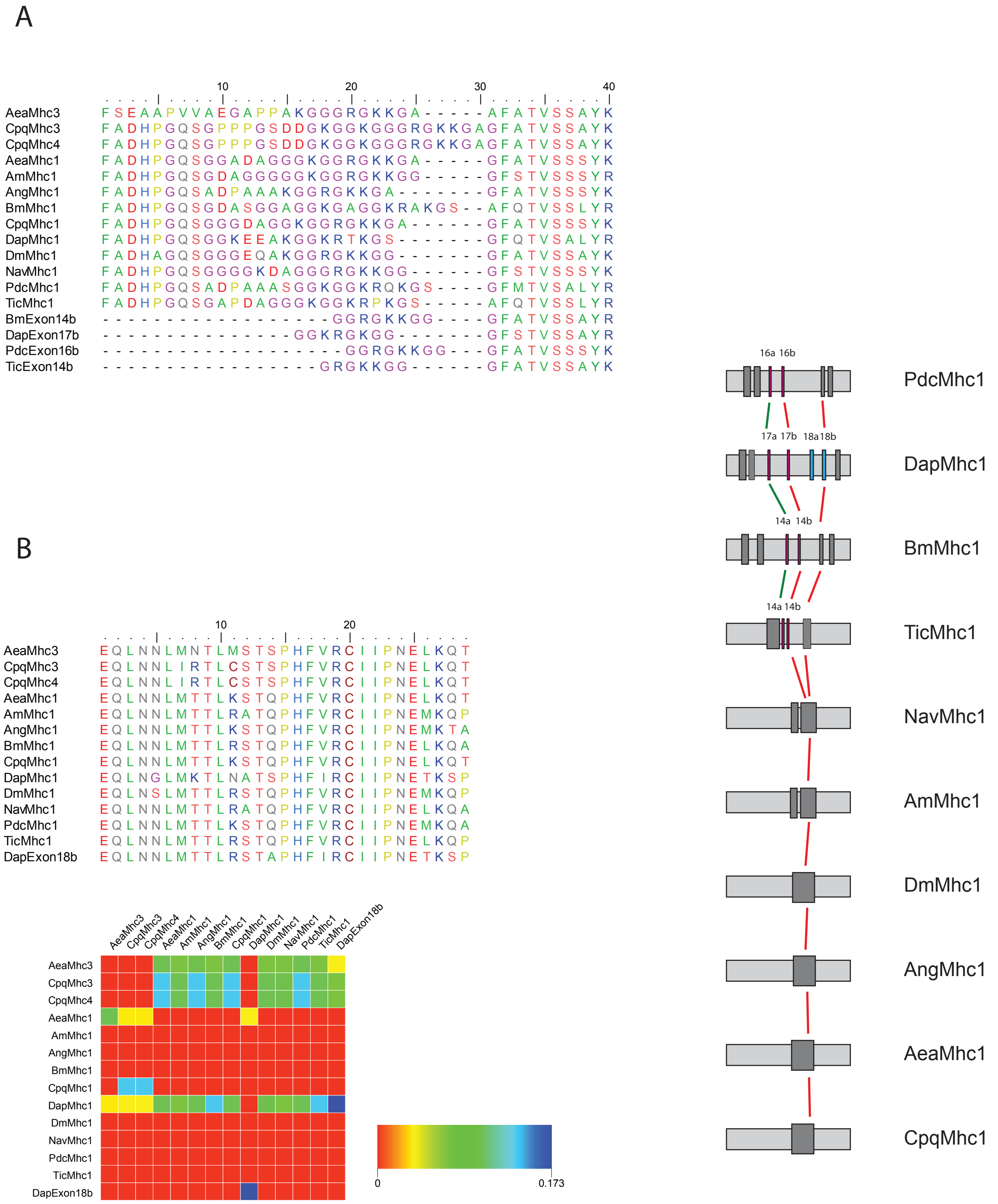
A
10 20 30 40
AeaMhc3 FSEAAPVVAEGAPPAKGGGRGKKGA------AFATVSSAYK
CpqMhc3 FADHPGQSGPPPGSDDGKGGKGGGRGKKGAGFATVSSAYK
CpqMhc4 FADHPGQSGPPPGSDDGKGGKGGGRGKKGAGFATVSSAYK
AeaMhc1 FADHPGQSGGADAGGGKGGRGKKGA------GFATVSSSYK
AmMhc1 FADHPGQSGDAGGGGGKGGRGKKGG------GFSTVSSSYR
AngMhc1 FADHPGQSADPAAAKGGRGKKGA--------GFATVSSSYK
BmMhc1 FADHPGQSGDASGGAGGKGAGGKRAKGS--AFQTVSSLYR
CpqMhc1 FADHPGQSGGGDAGGKGGRGKKGA-------GFATVSSSYK
DapMhc1 FADHPGQSGGKEEAKGGKRTKGS--------GFQTVSALYR
DmMhc1 FADHAGQSGGGEQAKGGRGKKGG--------GFATVSSAYK
NavMhc1 FADHPGQSGGGGKDAGGGRGKKGG-------GFSTVSSSYK
PdcMhc1 FADHPGQSADPAAASGGKGGKRQKGS----GFMTVSALYR
TicMhc1 FADHPGQSGAPDAGGGKGGKRPKGS-----AFQTVSSLYR
BmExon14b ------------------GGRGKKGG----GFATVSSAYR
DapExon17b ---------------GGKRGKGG--------GFSTVSSAYR
PdcExon16b -------------------GGRGKKGG---GFATVSSSYK
TicExon14b -----------------GRGKKGG-------GFATVSSAYK
B
10 20
AeaMhc3 EQLNNLMNTLMSTSPHFVRCIIPNELKQT
CpqMhc3 EQLNNLIRTLCSTSPHFVRCIIPNELKQT
CpqMhc4 EQLNNLIRTLCSTSPHFVRCIIPNELKQT
AeaMhc1 EQLNNLMTTLKSTQPHFVRCIIPNELKQT
AmMhc1 EQLNNLMTTLRATQPHFVRCIIPNEMKQP
AngMhc1 EQLNNLMTTLKSTQPHFVRCIIPNEMKTA
BmMhc1 EQLNNLMTTLRSTQPHFVRCIIPNELKQA
CpqMhc1 EQLNNLMTTLKSTQPHFVRCIIPNELKQT
DapMhc1 EQLNGLMKTLNATSPHFIRCIIPNETKSP
DmMhc1 EQLNSLMTTLRSTQPHFVRCIIPNEMKQP
NavMhc1 EQLNNLMTTLRATQPHFVRCIIPNELKQA
PdcMhc1 EQLNNLMTTLKSTQPHFVRCIIPNEMKQA
TicMhc1 EQLNNLMTTLRSTQPHFVRCIIPNELKQP
DapExon18b EQLNNLMTTLRSTAPHFIRCIIPNETKSP
AeaMhc3 CpqMhc3 CpqMhc4 AeaMhc1 AmMhc1 AngMhc1 BmMhc1 CpqMhc1 DapMhc1 DmMhc1 NavMhc1 PdcMhc1 TicMhc1 DapExon18b
AeaMhc3
CpqMhc3
CpqMhc4
AeaMhc1
AmMhc1
AngMhc1
BmMhc1
CpqMhc1
DapMhc1
DmMhc1
NavMhc1
PdcMhc1
TicMhc1
DapExon18b
0
0.173
16a 16b
PdcMhc1
17a 17b 18a 18b
DapMhc1
14a 14b
BmMhc1
14a 14b
TicMhc1
NavMhc1
AmMhc1
DmMhc1
AngMhc1
AeaMhc1
CpqMhc1

Figure 7

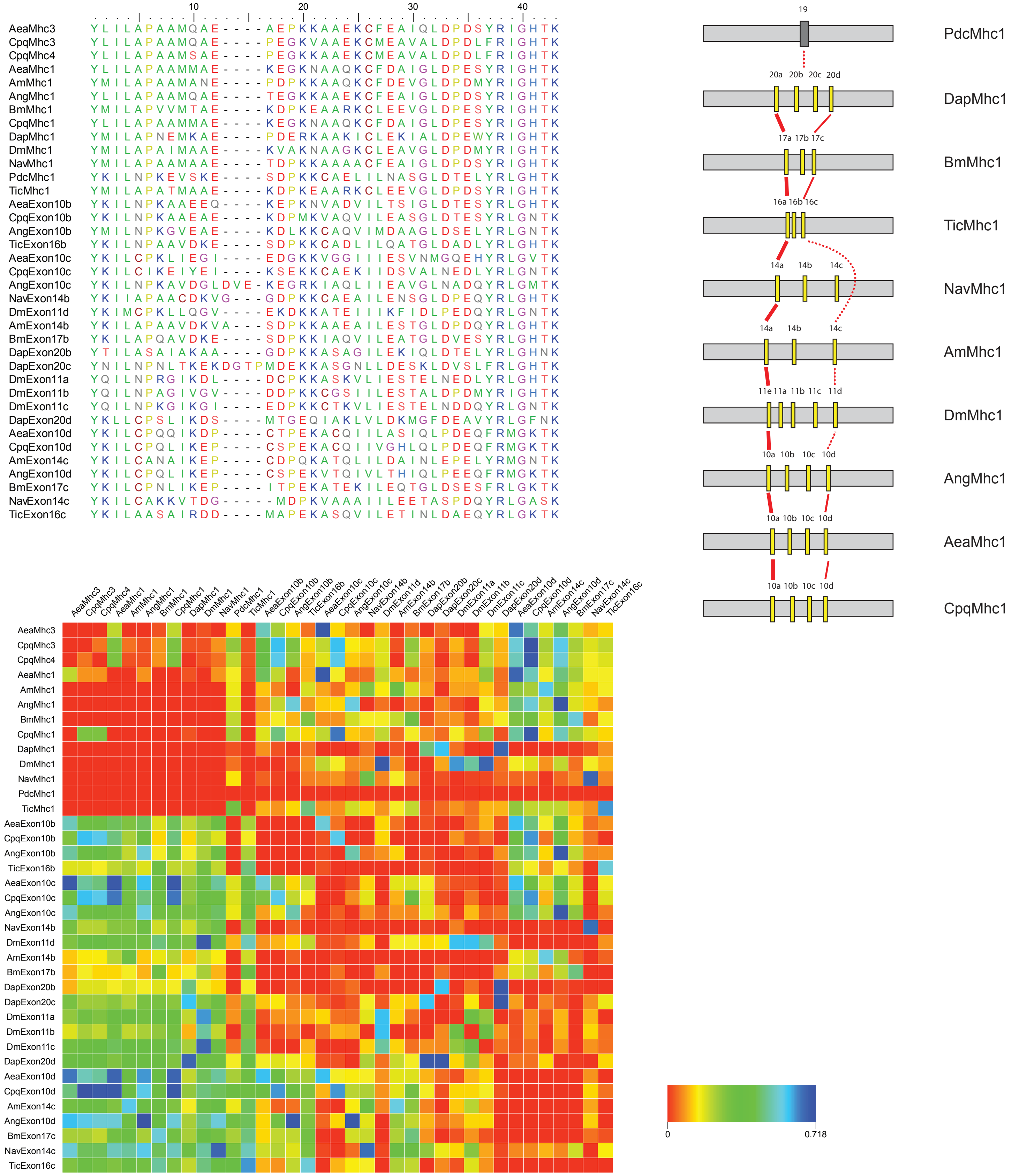
10
20
30
40
AeaMhc3 YLILAPAAMQAE----AEPKKAAEKCFEAIQLDPDSYRIGHTK
CpqMhc3 YLILAPAAMQAE----PEGKVAAEKCMEAVALDPDLFRIGHTK
CpqMhc4 YLILAPAAMSAE----PEGKKAAEKCMEAVALDPDLFRIGHTK
AeaMhc1 YLILAPAAMMAE----KEGKNAAQKCFDAIGLDPESYRIGHTK
AmMhc1 YMILAPAAMANE----PDPKKAAQKCFDEVGLDPDMYRIGHTK
AngMhc1 YLILAPAAMQAE----TEGKKAAEKCFEAIGLDPDSYRIGHTK
BmMhc1 YMILAPVVMTAE----KDPKEAARKCLEEVGLDPESYRIGHTK
CpqMhc1 YLILAPAAMMAE----KEGKNAAQKCFDAIGLDPESYRIGHTK
DapMhc1 YMILAPNEMKAE----PDERKAAKICLEKIALDPEWYRIGHTK
DmMhc1 YMILAPAIMAAE----KVAKNAAGKCLEAVGLDPDMYRIGHTK
NavMhc1 YMILAPAAMAAE----TDPKKAAAACFEAIGLDPDSYRIGHTK
PdcMhc1 YKILNPKEVSKE----SDPKKCAELILNASGLDTELYRLGHTK
TicMhc1 YMILAPATMAAE----KDPKEAARKCLEEVGLDPDSYRIGHTK
AeaExon10b YKILNPKAAEEQ----KEPKNVADVILTSIGLDTESYRLGHTK
CpqExon10b YKILNPKAAEAE----KDPMKVAQVILEASGLDTESYRLGNTK
AngExon10b YMILNPKGVEAE----KDLKKCAQVIMDAAGLDSELYRLGNTK
TicExon16b YKILNPAAVDKE----SDPKKCADLILQATGLDADLYRLGHTK
AeaExon10c YKILCPKLIEGI----EDGKKVGGIIIESVNMGQEHYRLGVTK
CpqExon10c YKILCIKEIYEI----KSEKKCAEKIIDSVALNEDLYRLGNTK
AngExon10c YKILNPKAVDGLDVE-KEGRKIAQLIIEAVGLNADQYRLGMTK
NavExon14b YKIIAPAACDKVG---GDPKKCAEAILENSGLDPEQYRLGHTK
DmExon11d YKIMCPKLLQGV----EKDKKATEIIIKFIDLPEDQYRLGNTK
AmExon14b YKILAPAAVDKVA---SDPKKAAEAILESTGLDPDQYRLGHTK
BmExon17b YKILAPQAVDKE----SDPKKIAQVILEATGLDVESYRLGHTK
DapExon20b YTILASAIAKAA----GDPKKASAGILEKIQLDTELYRLGHNK
DapExon20c YNILNPNLTKEKDGTPMDEKKASGNLLDESKLDVSLFRLGHTK
DmExon11a YQILNPRGIKDL----DCPKKASKVLIESTELNEDLYRLGHTK
DmExon11b YQILNPAGIVGV----DDPKKCGSIILESTALDPDMYRIGHTK
DmExon11c YQILNPKGIKGI----EDPKKCTKVLIESTELNDDQYRLGNTK
DapExon20d YKLLCPSLIKDS----MTGEQIAKLVLDKMGFDEAVYRLGFNK
AeaExon10d YKILCPQQIKDP----CTPEKACQIILASIQLPDEQFRMGKTK
CpqExon10d YKILCPQLIKEP----CSPEKACQIIVGHLQLPDEQFRMGKTK
AmExon14c YKILCANAIKEP----CDPQKATQLILDAINLEPELYRMGNTK
AngExon10d YKILCPQLIKEP----CSPEKVTQIVLTHIQLPEEQFRMGKTK
BmExon17c YKILCPNLIKEP----ITPEKATEKILEQTGLDSESFRLGKTK
NavExon14c YKILCAKKVTDG-----MDPKVAAAIILEETASPDQYRLGASK
TicExon16c YKILAASAIRDD----MAPEKASQVILETINLDAEQYRLGKTK
19
PdcMhc1
20a 20b 20c 20d
DapMhc1
17a 17b 17c
BmMhc1
16a 16b 16c
TicMhc1
14a 14b 14c
NavMhc1
14a 14b 14c
AmMhc1
11e 11a 11b 11c 11d
DmMhc1
10a 10b 10c 10d
AngMhc1
10a 10b 10c 10d
AeaMhc1
10a 10b 10c 10d
CpqMhc1
AeaMhc3
CpqMhc3
CpqMhc4
AeaMhc1
AmMhc1
AngMhc1
BmMhc1
CpqMhc1
DapMhc1
DmMhc1
NavMhc1
PdcMhc1
TicMhc1
AeaExon10b
CpqExon10b
AngExon10b
TicExon16b
AeaExon10c
CpqExon10c
AngExon10c
NavExon14b
DmExon11d
AmExon14b
BmExon17b
DapExon20b
DapExon20c
DmExon11a
DmExon11b
DmExon11c
DapExon20d
AeaExon10d
CpqExon10d
AmExon14c
AngExon10d
BmExon17c
NavExon14c
TicExon16c
0
0.718

Figure 8

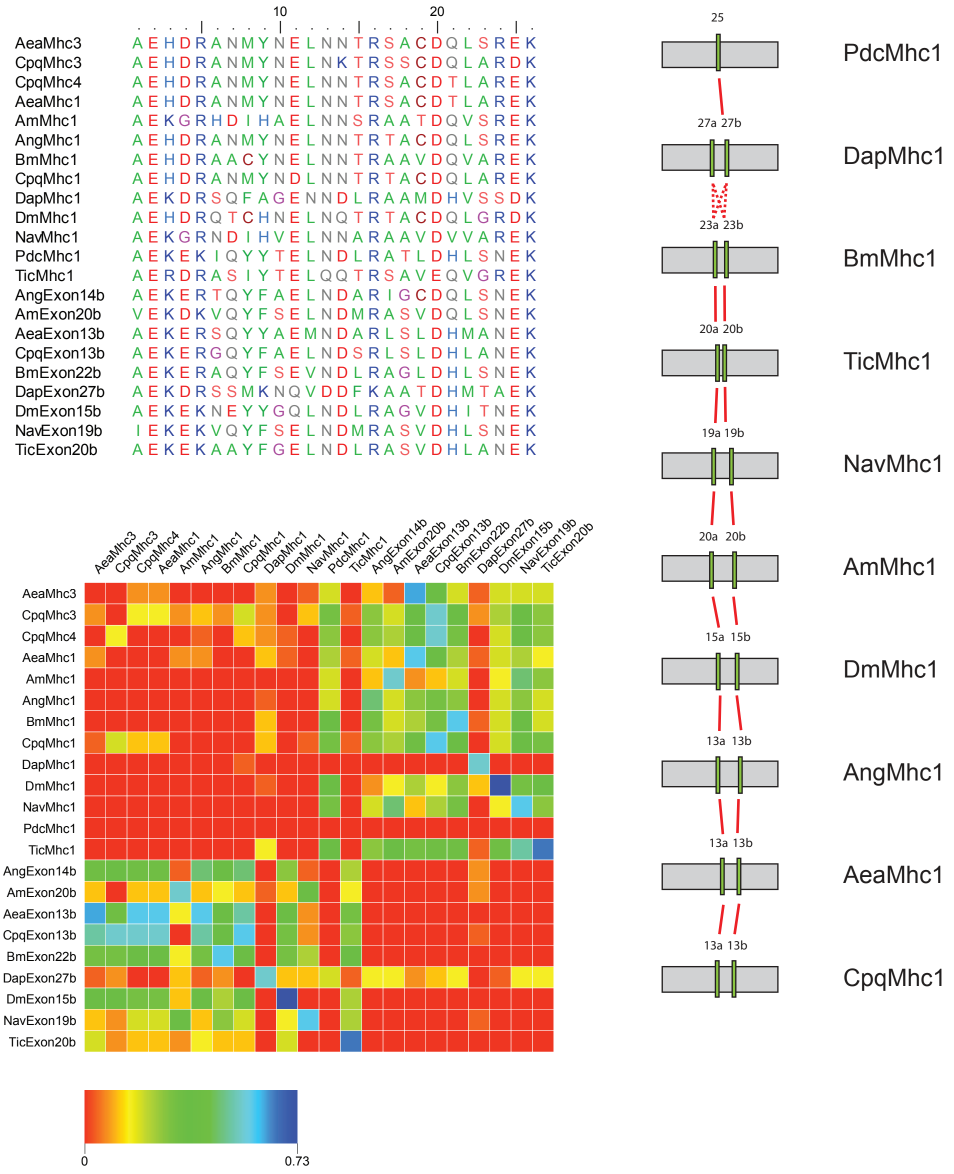

10
20
AeaMhc3 AEHDRANMYNELNNTRSACDQLSREK
CpqMhc3 AEHDRANMYNELNKTRSSCDQLARDK
CpqMhc4 AEHDRANMYNELNNTRSACDTLAREK
AeaMhc1 AEHDRANMYNELNNTRSACDTLAREK
AmMhc1 AEKGRHDIHAELNNSRAATDQVSREK
AngMhc1 AEHDRANMYNELNNTRTACDQLSREK
BmMhc1 AEHDRAACYNELNNTRAAVDQVAREK
CpqMhc1 AEHDRANMYNDLNNTRTACDQLAREK
DapMhc1 AEKDRSQFAGENNDLRAAMDHVSSDK
DmMhc1 AEHDRQTCHNELNQTRTACDQLGRDK
NavMhc1 AEKGRNDIHVELNNARAAVDVVAREK
PdcMhc1 AEKEKIQYYTELNDLRATLDHLSNEK
TicMhc1 AERDRASIYTELQQTRSAVEQVGREK
AngExon14b AEKERTQYFAELNDARIGCDQLSNEK
AmExon20b VEKDKVQYFSELNDMRASVDQLSNEK
AeaExon13b AEKERSQYYAEMNDARLSLDHMANEK
CpqExon13b AEKERGQYFAELNDSRLSLDHLANEK
BmExon22b AEKERAQYFSEVNDLRAGLDHLSNEK
DapExon27b AEKDRSSMKNQVDDFKAATDHMTAEK
DmExon15b AEKEKNEYYGQLNDLRAGVDHITNEK
NavExon19b IEKEKVQYFSELNDMRASVDHLSNEK
TicExon20b AEKEKAAYFGELNDLRASVDHLANEK
AeaMhc3 CpqMhc3 CpqMhc4 AeaMhc1 AmMhc1 AngMhc1 BmMhc1 CpqMhc1 DapMhc1 DmMhc1 NavMhc1 PdcMhc1 TicMhc1 AngExon14b AmExon20b AeaExon13b CpqExon13b BmExon22b DapExon27b DmExon15b NavExon19b TicExon20b
0
0.73
25
PdcMhc1
27a 27b
DapMhc1
23a 23b
BmMhc1
20a 20b
TicMhc1
19a 19b
NavMhc1
20a 20b
AmMhc1
15a 15b
DmMhc1
13a 13b
AngMhc1
13a 13b
AeaMhc1
13a 13b
CpqMhc1

Figure 9

```
                   10        20        30        40
          ....|....|....|....|....|....|....|....|..
AeaMhc3   RGGSAQRGGSPALSTV*
AeaExon16 RAGSVQRGASPA-PSVIRA*
AeaExon17 RAGSVQRGASPA-PQRQPSVMPGLAGLNFPTFDDHGF*
AngExon15 RAGSVQRGASPAVSTM*
AngExon16 RAGSVQRGASPA-PSVVRA*
AngExon17 RAGSVQRGASPA-PQRQPSAMPALAGLNLPTFDDHGF*
AmExon26  RGGSAARGLSPPA-PQQKARKAPSALE*
AmExon27  RGGSAARGLSPPA-PHRPAFKPQLDGSAFPPRFDLQPDGEL*
BmExon28  RAGSAARGVSPA-ASIKGRP*
BmExon29  RAGSAARGVSPA-PQRSRPALADGFGTFPPRFDLAPEDF*
CpqMhc3   ASSTAQSGGGGSAR*
CpqMhc4   RGGSAGRGASPAVSTI*
CpqExon16 RAGSVQRGASPA-PSVIRA*
CpqExon17 RAGSVQRGASPA-PQRQSAMPSLAALGLPTFDDHAF*
DapExon30 RGGSASR-LSPP-PQMKPRSKRDFE*
DmExon18  RAGSVGRGASPA-I*
DmExon19  RAGSVGRGASPA-PRATSVRPQFDGLAFPPRFDLAPENEF*
NavMhc1   RGGSAARGISPA-PHRPVNRPQFDGSAFPPRFDLMPDGL*
PdcExon33 RAGSAPRALSPA-PPQNRSRLALE*
PdcExon34 RAGSAPRALSPA-HHRPPRPQLDGMMAFPPRFDFHPEGEL*
TicExon22 RGGSVARGGSPA-PPRQRPQMDGLTFPPRFDLAPDEI*
```
